# Test development, optimization and validation of a WGS pipeline for genetic disorders

**DOI:** 10.1186/s12920-023-01495-x

**Published:** 2023-04-05

**Authors:** Ziying Yang, Xu Yang, Yan Sun, Yaoshen Wang, Lijie Song, Zhihong Qiao, Zhonghai Fang, Zhonghua Wang, Lipei Liu, Yunmei Chen, Saiying Yan, Xueqin Guo, Junqing Zhang, Chunna Fan, Fengxia Liu, Zhiyu Peng, Huanhuan Peng, Jun Sun, Wei Chen

**Affiliations:** 1 College of Life Sciences, University of Chinese Academy of Sciences, Beijing, 100049 China; 2 Tianjin Medical Laboratory, BGI-Tianjin, BGI-Shenzhen, Tianjin, 300308 China; 3 BGI-Tianjin, BGI-Shenzhen, Tianjin, 300308 China; 4 Department of Paediatrics, Pu’er People’s Hospital, Pu’er, 665000 China; 5 BGI Genomics, BGI-Shenzhen, Shenzhen, 518083 China; 6 DTU Bioengineering, Technical University of Denmark, 2800 Kongens Lyngby, Denmark; 7 BGI-Wuhan Clinical Laboratories, BGI-Shenzhen, Wuhan, 430074 China; 8 Clinical Laboratory of BGI Health, BGI-Shenzhen, Shenzhen, 518083 China; 9 Pu’er People’s Hospital, Pu’er, 665000 China

**Keywords:** Whole genome sequencing, Genetic disorders, Clinical diagnosis, Bioinformatics pipelines

## Abstract

**Background:**

With advances in massive parallel sequencing (MPS) technology, whole-genome sequencing (WGS) has gradually evolved into the first-tier diagnostic test for genetic disorders. However, deployment practice and pipeline testing for clinical WGS are lacking.

**Methods:**

In this study, we introduced a whole WGS pipeline for genetic disorders, which included the entire process from obtaining a sample to clinical reporting. All samples that underwent WGS were constructed using polymerase chain reaction (PCR)-free library preparation protocols and sequenced on the MGISEQ-2000 platform. Bioinformatics pipelines were developed for the simultaneous detection of various types of variants, including single nucleotide variants (SNVs), insertions and deletions (indels), copy number variants (CNVs) and balanced rearrangements, mitochondrial (MT) variants, and other complex variants such as repeat expansion, pseudogenes and absence of heterozygosity (AOH). A semiautomatic pipeline was developed for the interpretation of potential SNVs and CNVs. Forty-five samples (including 14 positive commercially available samples, 23 laboratory-held positive cell lines and 8 clinical cases) with known variants were used to validate the whole pipeline.

**Results:**

In this study, a whole WGS pipeline for genetic disorders was developed and optimized. Forty-five samples with known variants (6 with SNVs and Indels, 3 with MT variants, 5 with aneuploidies, 1 with triploidy, 23 with CNVs, 5 with balanced rearrangements, 2 with repeat expansions, 1 with AOHs, and 1 with exon 7–8 deletion of *SMN1* gene) validated the effectiveness of our pipeline.

**Conclusions:**

This study has been piloted in test development, optimization, and validation of the WGS pipeline for genetic disorders. A set of best practices were recommended using our pipeline, along with a dataset of positive samples for benchmarking.

**Supplementary Information:**

The online version contains supplementary material available at 10.1186/s12920-023-01495-x.

## Introduction

With advances in MPS technology, WGS has gradually been established as the first-tier diagnostic test for genetic disorders [1–3]. WGS possesses the potential to detect nearly all forms of genetic variation simultaneously [4]. As a primary clinical test, WGS provides greater diagnostic yield than conventional genetic testing [1, 6]. Although compared to other testing method, WGS remains very expensive and has limitations in accuracy for variants in regions of high homology, low complexity, and other technically challenging regions [5], WGS as a primary clinical test has demonstrated diagnostic superiority compared with conventional testing in pediatric patients [5, 6] and critically ill infants [7, 8]. Recently, an increasing number of laboratories have set up their own pipelines for clinical WGS.

WGS follows basic steps, including template preparation, library construction, MPS, and bioinformatics analysis. With the booming of clinical WGS over the past few years, researchers have recognized that finding consistent results among different laboratories is often difficult due to variations in sequencing platforms and a wide range of analysis pipelines. Factors influencing template preparation, library construction, sequencing, and bioinformatics analysis may influence the results of clinical WGS [9], resulting in different results among laboratories. For example, the analytical performance for CNV detection varied immensely using different calling tools [10]. To solve this problem, benchmarking resources, best practices and recommendations are needed for labs when introducing WGS into clinical practice [3, 11, 12]. However, deployment practice and testing of pipelines for clinical WGS are lacking.

In this study, we developed an optimized WGS pipeline for genetic disorders. Through the deployment of our WGS pipeline, we first present recommended strategies, tools, and resources for the detection of different types of variants. We did not explore all of the factors that may influence WGS results, but rather focused on the test development, detection and validation of some variant types that have not been well established. Subsequently, using 45 samples with various classes of known variants, we tested the performance of our WGS pipeline for the detection of various variants. This study piloted the test development, optimization, and validation of the WGS pipeline for genetic disorders and provides a reference for clinical WGS.

## Materials and methods

### Workflow of WGS

**Fig. 1:**
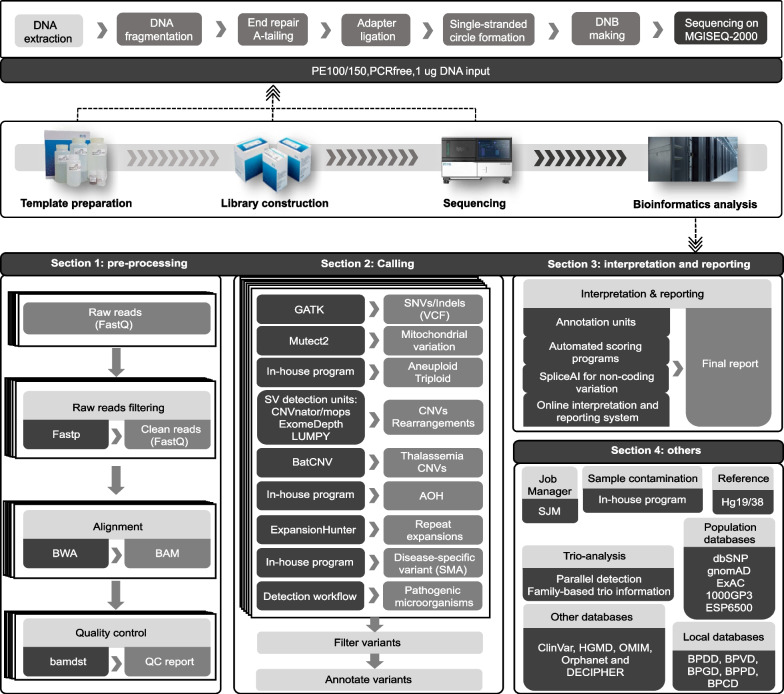
Study design

The entire workflow for WGS is presented in Fig. 1. All samples that underwent WGS were constructed using PCR-free library preparation protocols and sequenced on the MGISEQ-2000 platform. In general, DNA of reference samples (Coriell, Camden, NJ) and clinical samples with known variants (Additional file 1: Table S1) were first extracted using the MagPure Tissue & Blood DNA KF Kit (Magen, China). Subsequently, one microgram of DNA was used to generate paired end reads of 150 bp/100 bp according to the PCR-free library preparation protocols and sequencing protocols [13]. The bioinformatics analysis pipeline included 4 sections for the detection of potential variants (Fig. 1). Recommended strategies, tools, and resources were provided during test development and deployment of the entire process. Fourteen commercially available positive samples, 23 laboratory-held positive cell lines and 8 clinical cases with various classes of known variants were used to evaluate and optimize the performance of variant calling.

### Library construction and sequencing

Some parameters (such as DNA input, read length and library preparation) may affect the performance of WGS and ultimately influence variation detection sensitivity. Our previous reports showed that PCR-free WGS with 1 µg DNA input exhibited a better depth of coverage and genotype quality distribution than PCR-based WGS with differing DNA inputs [13]. With respect to the depth of coverage (DP), a mean depth of 30–50X is the most widely used mean DP for WGS [6, 14]. Our previous reports also showed that the current standard of the demonstrated mean depth of 40X may be sufficient for SNV/indel detection and identification of most CNVs. Based on these investigations and considerations of cost-effectiveness, we used a mean DP of ~ 40X for WGS (PCR-free) to investigate the recommended strategies, tools, and resources for the detection of different types of variants in this study.

In general, WGS of all samples was performed with a modified protocol using the MGISEQ-2000 platform [13]. First, the quality of the genomic DNA for all the samples was assessed using Qubit (Thermo Fisher Scientific) and gel electrophoresis. Next, 1 µg DNA for each sample was used to build a DNA fragment library using segmentase (MGI Tech Co., Ltd., BGI). After end repair and A-tailing, DNA fragments were ligated to the adapter sequence. Following purification, samples were—subjected to the following circularization process. After making DNA NanoBalls (DNBs), the DNBs were loaded into a sequencing chip for pair-end (150 bp/100 bp) sequencing (MGISEQ-2000RS High-throughput sequencing kit, PE150/PE100, V3.0, MGI Tech Co., Ltd.) on the MGISEQ-2000 platform. For a case, the average sequencing time required was ~ 36 h for PE100 and ~ 45 h for PE150. FASTQ data were subsequently generated for further bioinformatics analysis.

### Bioinformatics pipeline

The implementation of a standardized bioinformatics pipeline is essential for robust detection of various types of variants, which requires careful attention to test development, combination, optimization and validation of various tools. Here, we developed an optimized bioinformatics pipeline for WGS. During the deployment of our pipeline, we identify recommended tools and resources for the detection of different types of variants. The entire bioinformatics pipeline can be mainly divided into 4 sections: preprocessing, calling, interpretation and reporting, and others (Fig. 1).

## Section 1: preprocessing

### Fastp: raw reads filtering

For the first step in the bioinformatics pipeline, plenty of tools that are available to remove adapter sequences and filter raw reads. Here, we used fastp (version 0.20.0) [15] for raw read filtering (Fig. 1). Fastp takes raw reads and the adapter sequences as input, and outputs clean reads according to user defined parameters. In addition to the removal of adapter sequences, reads that met at least one of the following conditions were filtered out: (1) proportion of N bases ≥ 10%; (2) proportion of bases with low quality values (quality value < 5) ≥ 50%; and (3) the average quality value < 10.

### BWA: FastQ to BAM

Alignment is one of the most critical steps in a bioinformatics pipeline and, refers to the mapping of the sequencing results to a reference genome. The Genome Analysis Toolkit (GATK) Best Practices workflow [12] recommends the Burrows-Wheeler Aligner (BWA) [16] as a robust read mapping algorithm that can be used to align the sequence of tested samples to a reference sequence. The BWA, is the most widely used tool for alignment of MPS data. Here, we also used BWA (version 0.7.17-r1188) for alignment. Using BWA, clean reads obtained in the previous step were aligned to the human reference sequence to generate mapping results in the BAM format. Subsequently, the BAM file could be used as input for calling tools (such as GATK and CNVnator).

### Bamdst: quality control

DP is a crucial factor in routine quality control (QC) of MPS results. To verify that sufficient sequencing data has been collected, most centers using MPS technology would determine thresholds for average DP that must be achieved for a certain fraction of target bases [17]. Here, we used bamdst (version 1.0.5) (https://github.com/shiquan/bamdst), a lightweight tool, for routine QC (Fig. 1). Bamdst takes a sorted BAM file and target region file as input. The output of bamdst includes coverage information and plots of the target and flank regions.

### ***Section 2: calling***

The potential of WGS to detect all types of variants relies heavily on calling tools. In this study, we describe a suite of tools, that can detect various types of variants simultaneously, including SNVs, indels, structural variants (CNVs and balanced rearrangements), MT variants, and other complex variants (repeat expansions, pseudogenes and AOHs).

### GATK: BAM to VCF

Researchers at the Broad Institute developed a best-practice workflow (BWA for alignment and GATK for variant calling) for the detection of variations [12] that, is one of the most commonly used and well-accepted workflows for the detection of SNVs and indels. Here, we also used GATK (version 4.0.11.0) for the detection of SNVs and Indels. With the aid of sambamba (version 0.6.8) and MarkDuplicates from GATK (version 4.0.11.0), the BAM file obtained in the previous step is used as input to generate calling results in the VCF format. The VCF file can be used as input for further filtering and annotation.

### Mutect2: BAM to VCF

We implemented Mutect2 from GATK (version 4.0.11.0) [18] for the detection of MT variants. Mutect2 requires a BAM file for input. The output of Mutect2 includes the detected MT variants in the format VCF format.

### Aneuploidy and triploidy detection program

We developed an in-house program for the detection of aneuploidies and triploidies. This program takes the ratio of all the heterozygous SNPs as input. The output includes plots of the SNP ratio and the status (triploidy, diploid or aneuploidy) of each chromosome.

### SV detection units: CNVnator/mops, exomedepth and LUMPY

We implemented an SV detection suite in our pipeline for the detection of SVs, including CNVnator/mops (version 0.3.2) [19], the “ExomeDepth” module of R (version 3.6.2) [20] and LUMPY (version 0.2.13) [21]. We also used LUMPY for the detection of other types of SVs, including balanced translocations and inversions. CNVnator, ExomeDepth and LUMPY take BAM files as input, and output CNV results in different formats. According to the output of different tools, we developed our own scripts to gather all the results for filtering and annotation.

### BatCNV

BatCNV is a self-developed tool for the detection of CNVs in thalassemia [22]. BatCNV takes a BAM file as input. The output of BatCNV includes plots and common thalassemia genotypes.

### AOH detection program

We used the “homozyg” module of PLINK (version 1.90b6.12) [23] for the detection of AOH. This program takes the variant allele fraction (VAF) of SNPs as input. The output includes identified homozygous regions and the proportion of homozygous sites.

### ExpansionHunter

We used ExpansionHunter (version 3.2.0) [24] for the detection of repeat expansion. ExpansionHunter takes the BAM file as input. The output of ExpansionHunter includes variant genotypes and other useful information in the format of VCF.

### Disease-specific variant detection program for SMA

We developed an in-house program specifically for the detection of spinal muscular atrophy (SMA). This program takes the depth of exons and SNPs (generated using GATK’s DepthOfCoverage analysis tool) of each sample as input. The output includes the copy number of the *SMN1* and *SMN2* genes.

### Detection workflow of pathogenic microorganisms

We developed and included a workflow for the detection of pathogenic microorganisms, which is implemented in the WGS pipeline. This workflow involves bacterial read separation using KneadData (version 0.10.0) (https://bitbucket.org/biobakery/kneaddata/wiki/Home) and detecting pathogenic microorganisms using MetaPhlAn (version 3.0.13) [25], Kraken (version 2.1.2) [26] and Bracken (version 2.5) [27]. Reads that do not map anywhere on the human reference genome from a BAM file are extracted to generate a FASTQ file. This workflow takes the FASTQ files as input. The output includes the detected microbial communities and relative abundance.

## Section 3: interpretation and reporting

### Annotation units

The results of certain tools are often difficult to interpret. Annotation is necessary to facilitate interpretation and accelerate diagnosis. In our pipeline, we developed a suite of annotation programs for SNVs, Indels, CNVs, balanced translocations and inversions, AOHs, and repeat expansions.

### Automated scoring programs

To further facilitate the interpretation process, we implemented autoPVS1 [28] and AutoCNV (version 1.1.0) [29] to classify and generate predictions for SNVs and CNVs, respectively. AutoPVS1 is an automatic classification tool for PVS1 interpretation of null variants. AutoCNV is a semiautomatic CNV interpretation system based on the 2019 ACMG/ClinGen Technical Standards for CNVs. AutoPVS1 and AutoCNV can accelerate and facilitate the interpretation of SNVs and CNVs.

### SpliceAI

We used SpliceAI (version 1.3) [30], a deep learning-based tool, to identify splice variants. SpliceAI takes a VCF file as input and outputs predictions for variants within genes in the VCF format.

### Online interpretation and reporting system

We also developed a laboratory-held web-based system for interpretation and reporting (https://genetics.bgidx.cn/). This system provides a more user-friendly method for interpretation, especially for clinical scientists. After uploading the results from previous steps, the server provides detailed criteria and supportive evidence for all the variants after the analysis. The server generates a final report for all of the variants. Clinical scientists can then download the report for genetic counseling.

### ***Section 4: others***

#### SJM

We used Simple Job Manager (SJM) (version 1.2.0) to manage our jobs. SJM provides a convenient method for specifying dependencies between jobs and the resource requirements for each job (https://github.com/StanfordBioinformatics/SJM).

### Trio-analysis

A higher diagnostic yield has been reported for trio-analysis [10]. In our pipeline, trio-analysis was also developed, which could detect all variants in parallel. Trio-analysis offers several key advantages, including the ability to take advantage of family-based trio information. Using the segregation pattern, trio-analysis can help eliminate false-positives.

### Contamination QC

Maternal cell contamination presents a serious risk for misdiagnosis, especially for chorionic villus and amniotic fluid, which are frequently used in prenatal diagnosis. Based on VAF, we developed an in-house model that could detect contamination ratios > 5%. This model was used for contamination QC in our pipeline.

### Databases

The successful detection of potential variants cannot be achieved without the support of databases. We implemented dbSNP 147 [31], gnomAD (release 2.1) (https://gnomad.broadinstitute.org), Exome Aggregation Consortium (ExAC) (release 1), 1000 Genomes Project phase 3 (1000GP3, IGSR: The International Genome Sample Resource. https://www.internationalgenome.org/home) and NHLBI GO Exome Sequencing Project of 6500 (ESP6500) as a population-based polymorphism database. ClinVar [32], Human Gene Mutation Database (HGMD) [33], Online Mendelian Inheritance in Man (OMIM) [34], Orphanet [35] and DECIPHER (https://decipher.sanger.ac.uk/.) databases were also implemented in our pipeline. All the databases can be updated quarterly or yearly through our internal script. Based on publicly available databases and our own data, we also developed a series of our own databases (BPDD: BGI PHOENIX-Genetic Disease Database; BPVD: BGI PHOENIX-Variant Database; BPGD: BGI PHOENIX-Gene Database; BPPD: BGI PHOENIX-Phenotype Database; BPCD: BGI PHOENIX-in-house Control Database (WGS data of healthy Chinese population)) for annotation and interpretation.

### Analysis of 45 samples with known variants

In this study, 45 samples with known variants of various types were recruited and reanalyzed using our WGS pipeline. All the variants (Additional file 1: Table S1) were validated previously, including 6 SNVs and Indels, 3 MT variants, 5 aneuploidies, 1 triploidy, 23 CNVs, 5 balanced rearrangements, 2 repeat expansions, 1 sample with multiple AOHs, and 1 with exon 7–8 deletion of the *SMN1* gene.

The samples we recruited were all positive samples of various variants. For each variant type, samples with other variant types could be regarded as negative samples. Based on the definitions of sensitivity and specificity [36], taking samples with SNVs as an example, we calculated sensitivity and specificity using the following formulas:

$$Sensitivity_{SNV} = \frac{Samples\; carrying\; disease - associated\; SNVs\; detected\; by\; WGS}{{Samples\; carrying \;disease - associated\; SNVs\; detected\; by \;Sanger}}$$

$$Specificity_{SNV} = \frac{Samples\; carrying\; other \;disease - associated\; variants\; detected\; by \;WGS}{{Samples\; carrying \;other\; disease - associated\; variants\; detected \;by\; Sanger}}$$

## Results

### Variant detection

All types of variants that can be detected using our pipeline are summarized in Additional file 1: Table S1, including SNVs, indels, structural variants (CNV and balanced rearrangement), MT variants, other complex variants (repeat expansion, pseudogene and AOH), and pathogenic microorganisms. Here, we recommend a suite of tools for calling various types of variants.

### SNV/Indels

In our pipeline, the best-practice workflow developed by the Broad Institute (BWA for alignment and GATK for variant calling) was used for the detection of SNV/Indels.

Six samples with known SNVs and Indels were used for validation (Additional file 1: Table S1). All 6 variants were confirmed previously by Sanger sequencing. All 6 variants were also successfully detected using our WGS pipeline. This best-practice workflow is one of the most commonly used and well-accepted workflows for the detection of SNVs and indels, the results of the 6 variants further demonstrated its detection sensitivity.

### Noncoding variants

SpliceAI was implemented for the prediction of splice variants in our pipeline (Additional file 1: Figure S1). Using a deep residual neural network, the effect on splicing (splice donor, splice acceptor, or neither) of each position in a pre-mRNA transcript can be predicted using SpliceAI. For genes with a high proportion of splicing mutations, SpliceAI exhibited better performance for the prediction of splicing alterations, and then significantly assisted in diagnosis. During the deployment of our pipeline, we also tested the performance of SpliceAI for the detection of splice variants using variants derived from 2 published articles [37, 38] and the HGMD database, (Additional file 1: Figure S1). Compared with MaxEntScan [39], scSNV [40] and MMSplice [41], SpliceAI exhibited better detection sensitivity, specificity, accuracy, and prediction rate for the prediction of splice-altering variants in the human genome (Additional file 1: Figure S1). Moreover, we observed that the prediction scope of MaxEntScan, scSNV and MMSplice is limited, which in turn further recommended SpliceAI for the detection of splice variants.

### MT variants

Mutect2 was implemented for the detection of MT variants in our pipeline. MT variants often exhibit a low allele fraction (AF), which may be easily mistaken for inherent sequencer noise. Mutect2 could provide high detection sensitivity for variants with low levels of AF and allow for the tracing of lineages of rare MT variants.

Three samples with known MT variants were used for validation (Additional file 1: Table S1). All 3 variants were confirmed previously by Sanger sequencing. All 3 variants were also successfully detected using our WGS pipeline (Additional file 1: Table S1). Mutect2 was widely used for somatic calling, and our results further demonstrated its ability to detect variants with extremely high depths. The heteroplasmy of the 3 variants was also provided by Mutect2.

### Aneuploidy and triploidy

In our pipeline, an in-house program was developed for the detection of aneuploidies and triploidies. This program was developed based on the mechanisms of diploid and triploidy. Diploid cells contain two copies of each autosomal chromosome; thus, theoretically, the ratio of all heterozygous SNPs should be close to 1/2. For triploid cells, the ratio of all heterozygous SNPs should be close to 1/3 or 2/3. In our program, binomial distribution was used to fit the ratio of all the heterozygous SNPs. Aneuploidy for each chromosome and triploidy can then be identified.

Six samples with known aneuploidies and triploidies were used for validation (Additional file 1: Table S1, Figures S2-S7). All variants were confirmed previously by karyotyping or chromosomal microarray analysis (CMA). All the aneuploidies and triploidies were also successfully detected using our WGS pipeline.

### CNV

CNVnator/mops, ExomeDepth and LUMPY were implemented in our pipeline for the detection of CNVs. Previous reports have demonstrated substantial variation in the sensitivity of CNV detection across different tools. Most CNV detection tools were developed based on read depth (read count) or read pair algorithms [42]. The combination of the results of two or more complementary tools may offer better detection sensitivity for CNVs with differing lengths. We implemented an SV detection suite in our pipeline. For large CNVs of more than 1 M, we recommended CNVnator, a depth-based CNV tool [10]. For other smaller CNVs, we recommended LUMPY, a CNV tool using both split and anomalous read pair information [10]. ExomeDepth, a depth-based CNV tool, is more suitable for the detection of exome CNVs.

Nineteen samples with known CNVs were used for validation (Additional file 1: Table S1). All -variants were confirmed previously by quantitative polymerase chain reaction (qPCR), low pass WGS, long range PCR, or CMA. All variants were also successfully detected using our WGS pipeline. After testing in our previously published article [10] and testing the nineteen samples with known CNVs in this study, we determined that the combination of the results of multiple complementary tools may offer superior detection sensitivity.

### Balanced translocation and inversion

LUMPY, a general probabilistic tool, was implemented in our pipeline for the detection of balanced translocations and inversions. LUMPY integrates read-pair, split-read and read-depth signals, and combines the information of sites of known variants to improve detection sensitivity. During the deployment of our pipeline, we also tested the performance of BreakDancer [43] and Delly [44] for the detection of balanced translocations and inversions. Compared with these tools, LUMPY was faster and exhibited better detection sensitivity for known and validated SVs in our lab (data not shown).

Four samples with known balanced translocations were used for validation (Additional file 1: Table S1, Figures S8-S12). All variants were confirmed previously by karyotyping. All variants were also successfully detected using our WGS pipeline.

### Thalassemia CNV

BatCNV, a self-developed tool, was implemented in our pipeline for the detection of CNVs in thalassemia. BatCNV was developed for the detection of large gene deletions and duplications. First, adjustable sliding windows in the genome (unique regions) were used to calculate the read count of each window. Then, GC correction and batch correction were applied to minimize data fluctuations. Subsequently, a hidden Markov model (HMM) algorithm was used to predict the copy number status of each window. Finally, thalassemia genotypes were determined by combining the breakpoint information. BatCNV largely reduced the influence of homology on the detection of thalassemia CNVs. The performance of BatCNV for the detection of CNVs in thalassemia has already been validated in a large-scale population in our previous reports [22, 45].

Four samples with known thalassemia CNVs were used for validation (Additional file 1: Table S1). All variants were confirmed previously by gap-PCR. All variants were also successfully detected using our WGS pipeline.

### AOH

In our pipeline, PLINK was used for the detection of AOH. Regions with AOH are indicated by a change in the rate of heterozygous SNPs and homozygous SNPs. Regions with terminal AOH ≥ 5 Mb were then reported. A literature survey indicated that several tools could detect AOH using NGS data, including UPDio and BCFtools/RoH. PLINK exhibited better detection sensitivity for known and validated AOHs in our lab (data not shown). In addition, using 101 regions with known AOHs, we determined the optimal window size (each window contains 20 SNPs) for PLINK in the detection of AOH (Additional file 1: methods).

One sample with multiple known AOHs was used for validation (Additional file 1: Table S1, Figures S13-S29). All AOHs were confirmed previously by CMA. Compared with the known variation verification method, our WGS pipeline detected a greater number of AOH regions. This is primarily because several AOH regions were split into subregions by PLINK. These results further demonstrated the ability of PLINK to detect AOH. We also determined the influence of window sizes on PLINK. We recommend determining the optimal window size for PLINK before using this method for AOH detection.

### Repeat expansion

To date, tools (using MPS data) for the detection of repeat expansion are still rare. ExpansionHunter, a sequence-graph based tool, was employed in our pipeline for the detection of repeat expansion. Expansions of short tandem repeats can cause several disorders, such as Fragile X Syndrome and Huntington's Disease. For long repeat regions, special algorithms should be developed to analyze short tandem repeats using MPS data.

Two samples with known repeat expansion (CTG/CAG) in the *DMPK* and *ATN1* genes were used for validation (Additional file 1: Table S1). All these variants were confirmed previously by PCR amplification and fragment analysis. Alleles larger than the read length are reported to be underestimated by ExpansionHunter [46]. Although ExpansionHunter was limited in the sizing of alleles considerably larger than the read length, all variants were also successfully detected using our WGS pipeline. Established limited tools aid in detecting repeat expansion, thus, ExpansionHunter was the first choice for our pipeline. However, based on the results of the two samples with known repeat expansion, we recommended that further analysis and additional validation methods should be performed for positive results detected by ExpansionHunter.

### SMA

In our pipeline, we developed an in-house program specifically for the detection of SMA [47]. More than 95% of SMA cases are caused by exon 7–8 deletion of the *SMN1* gene [48]. For the influence of *SMN2* (a homologous gene) on the detection of *SMN1* copy number, special algorithms should be developed using NGS data. In general, our in-house program calculated the copy number of *SMN1* and *SMN2* copy number based on the read number covering distinguished base pairs between *SMN1* and *SMN2*.

One sample with known copy number loss in exons 7–8 of the *SMN1* gene was used for validation (Additional file 1: Table S1). The variant was confirmed previously by multiplex ligation-dependent probe amplification (MLPA). The variant was also successfully detected using our WGS pipeline. This program has been validated in our previous studies and tested in a large cohort [47].

### Intrauterine infection

A pathogenic microorganism detection workflow was implemented in the WGS pipeline. In some circumstances, the detection of pathogenic microorganisms is crucial for WGS. For example, fetal structural anomalies caused by intrauterine infection may not be detectable by regular WGS. Our workflow was composed of a suite of well-known tools (KneadData, MetaPhlAn, Kraken 2 and Bracken) and its databases. Using this workflow, the tools applied in our workflow were easy to use and updated in a timely manner, and the databases used in our workflow were comprehensive and small in size.

During the process of deployment, we tested the performance of this workflow using virtual samples. Gradient dilutions of cytomegalovirus (CMV)-positive amniotic fluid and CMV-negative amniotic fluid were collected to prepare virtual amniotic fluid samples with CMV infection. After sequencing, artificially pooled amniotic fluid samples were tested using our workflow. The abundance of CMV nucleic acid was also determined by qPCR. The results revealed that our workflow could detect CMV reads at a rate of ~ 2.31 copies/ml, which was far more sensitive than qPCR.

### Analysis of 45 samples with known variants

For a certain variant type, samples with other variant types could be regarded as negative samples. For each variant type, the detection sensitivity and specificity of our WGS pipeline for the 45 samples was 100%. We manually interpreted the variants detected by the bioinformatics analysis pipeline. The numbers of variants and time required for manual interpretation of each type of variant for one sample are presented in Additional file 1: Table S2.

## Discussion

In this study, we developed an optimized WGS pipeline for genetic disorders. Focusing on test development, detection and validation of various variants that have not been well established, we presented recommended strategies, tools, and resources for the detection of different types of variants. We also tested the performance of our WGS pipeline for the detection of various variant types using 45 previously validated samples. Through this pilot study, we have achieved a deeper understanding of the capabilities and limitations of WGS for identifying and characterizing variants. Our study is particularly useful for clinical scientists to determine the range of sensitivities for different classes of variants for a particular WGS pipeline, which could be useful when interpreting and delivering clinical reports. The recommended strategies, tools, and resources presented here could also facilitate the introduction of WGS into clinical practice for clinical laboratories.

The WGS pipeline was optimized based on extensive literature research and testing. Some tools were selected based on published literature where best practices were demonstrated. For example, Van der Auwera GA published GATK best practices for SNVs/indels [12], which have been widely used. For the detection of repeat expansions, a limited number of tools have been reported. ExpansionHunter exhibited high performance based on our test. The selection of PLINK for the detection of AOHs was based on the testing of multiple tools. After testing, we found that PLINK was optimal (AOHs were detected in all enrolled positive samples). For CNV detection, our WGS pipeline employed 4 tools (CNVnator/mops, ExomeDepth and LUMPY) for the detection of CNVs with differing lengths. The combination of the results of multiple complementary tools may offer better detection sensitivity.

For testing a sample (taking 40X as an example) using our WGS pipeline, the cost primarily includes 4 parts: materials (reagents for DNA extraction, library construction and sequencing), labor, bioinformatics analysis (computing resources) and depreciation expenses. The cost of materials accounts for the largest proportion (51%). However, compared with other conventional testing methods, such as CMA, the cost of WGS remains much higher [49].

Clinical WGS is an ideal method for genetic disorders. However, WGS has some limitations. As clinical WGS has matured, we now have a much deeper understanding of the capabilities and limitations of WGS for identifying and characterizing variants. First, the potential of WGS for the detection of all types of variants highly relies on calling tools. Many tools have been developed with the development of WGS. However, the performance of different tools varies significantly. Standards addressing the definition and performance of a “best practice” tool for most classes of variants are lacking. Pilot studies in test development, optimization and validation of the WGS pipeline for genetic disorders are needed. One limitation of our study is that although we presented recommended strategies and tools in our WGS pipeline, we did not perform a comprehensive comparison of currently available tools for the detection of specific variants, which would be an interesting topic but remains out of the scope of this study. Second, although WGS possesses the potential to detect all kinds of variants, it does not mean that WGS is the only solution for a specific variant. For example, conventional molecular karyotyping is widely used to detect chromosomal abnormalities with CNVs of more than 5–10 Mb [50]. SNP arrays and CMA are widely used for the detection of SNVs and CNVs. Compared with these traditional methods, clinical WGS remains expensive for the detection of specific variants. Clinical scientists should choose the best method according to the patient’s clinical requirements. Here, clinical requirements not only refer to the phenotypes of the patient, but also include of whether the patient can afford to undergo the test. Third, with the increasing information generated by WGS, greater challenges arise for clinical scientists to interpret and transform raw sequencing data into timely diagnoses and positive diagnosis yields, and convenient annotation tools and automated scoring programs are needed to address these gaps. Moreover, even if WGS can detect many variant types, all variants should not necessarily be reported. Standards addressing the reporting criteria are lacking. Fourth, there were a limited number of testing samples with mosaicism, which is a limitation of this study. Therefore, the sensitivity in the detection of low-level mosaicism of variants in the nuclear and mitochondrial genomes cannot be provided in this pilot study.

We plan to test this system in other hospitals or clinical institutions, including experiments, sequencing, and bioinformatics analysis. For the bioinformatics analysis section, each section has been modularized and has application programming interfaces. A corresponding operating system is needed. For labs, taking a WGS sample with a production of 180 G bases as an example, the required peak storage for bioinformatics analysis is approximately 2 TB. If all bam files (separate bam files and the whole bam file) and VCF files are saved, 0.5 TB of storage space is needed. The hardware configuration requires 96-core and 384 G memory.

In this study, through the deployment of an optimized WGS pipeline for genetic disorders, we presented recommended strategies, tools, and resources for the detection of different types of variants. Using samples with known variants of various types, we validated and optimized the performance of our pipeline. This study piloted the test development, optimization, and validation of the WGS pipeline for genetic disorders and provided a set of recommendations and benchmarking resources for clinical WGS.

## Supplementary Information

**Additional file 1.** Supplementary Methods, Figures and Tables.

## Data Availability

The datasets of 14 commercially available positive samples used and analyzed during the current study have been deposited into CNGB Sequence Archive (CNSA) [51] of China National GeneBank DataBase (CNGBdb) [52] with accession number CNP0003651 (https://db.cngb.org/search/project/CNP0003651/). The other data generated and analyzed during the current study is not publicly available as they are patient samples (or cell line of patient samples) and sharing them could compromise research participant privacy.

## Abbreviations

MPS: Massive parallel sequencing
WGS: Whole-genome sequencing
PCR: Polymerase chain reaction
SNVs: Single nucleotide variants
indel: Insertions and deletions
CNVs: Copy number variants
AOH: Absence of heterozygosity
DP: Depth of coverage
DNBs: DNA nanoballs
GATK: Genome analysis toolkit
QC: Quality control
MT: Mitochondrial
VAF: Variant allele fraction
SMA: Spinal muscular atrophy
SJM: Simple job manager
ExAC: Exome aggregation consortium
1000GP3: 1000 Genomes project phase 3
ESP6500: Exome sequencing project of 6500
HGMD: Human gene mutation database
OMIM: Online mendelian inheritance in man
BPDD: BGI PHOENIX-genetic disease database
BPVD: BGI PHOENIX-variant database
BPGD: BGI PHOENIX-gene database
BPPD: BGI PHOENIX-phenotype database
BPCD: BGI PHOENIX-in-house control database
AF: Allele fraction
qPCR: Quantitative polymerase chain reaction
CMA: Chromosomal microarray analysis
HMM: Hidden Markov model
MLPA: Multiplex ligation-dependent probe amplification
CMV: Cytomegalovirus
CNSA: CNGB sequence archive
CNGBdb: China national genebank database

## References

[1] Lionel AC, Costain G, Monfared N, Walker S, Reuter MS, Hosseini SM (2018). Improved diagnostic yield compared with targeted gene sequencing panels suggests a role for whole-genome sequencing as a first-tier genetic test. Genet Med.

[2] Scocchia A, Wigby KM, Masser-Frye D, Del Campo M, Galarreta CI, Thorpe E (2019). Clinical whole genome sequencing as a first-tier test at a resource-limited dysmorphology clinic in Mexico. NPJ Genom Med.

[3] Marshall CR, Chowdhury S, Taft RJ, Lebo MS, Buchan JG, Harrison SM (2020). Best practices for the analytical validation of clinical whole-genome sequencing intended for the diagnosis of germline disease. NPJ Genom Med.

[4] Pang AW, MacDonald JR, Yuen RK, Hayes VM, Scherer SW (2014). Performance of high-throughput sequencing for the discovery of genetic variation across the complete size spectrum. G3: Genes Genomes Genetics.

[5] Clark MM, Stark Z, Farnaes L, Tan TY, White SM, Dimmock D (2018). Meta-analysis of the diagnostic and clinical utility of genome and exome sequencing and chromosomal microarray in children with suspected genetic diseases. NPJ Genom Med.

[6] Stavropoulos DJ, Merico D, Jobling R, Bowdin S, Monfared N, Thiruvahindrapuram B (2016). Whole genome sequencing expands diagnostic utility and improves clinical management in pediatric medicine. NPJ Genom Med.

[7] Farnaes L, Hildreth A, Sweeney NM, Clark MM, Chowdhury S, Nahas S (2018). Rapid whole-genome sequencing decreases infant morbidity and cost of hospitalization. NPJ Genom Med.

[8] Saunders CJ, Miller NA, Soden SE, Dinwiddie DL, Noll A, Alnadi NA (2012). Rapid whole-genome sequencing for genetic disease diagnosis in neonatal intensive care units. Sci Transl Med.

[9] Goodwin S, McPherson JD, McCombie WR (2016). Coming of age: ten years of next-generation sequencing technologies. Nat Rev Genet.

[10] Sun Y, Liu F, Fan C, Wang Y, Song L, Fang Z (2021). Characterizing sensitivity and coverage of clinical WGS as a diagnostic test for genetic disorders. BMC Med Genomics.

[11] Koboldt DC (2020). Best practices for variant calling in clinical sequencing. Genome Med.

[12] Van der Auwera GA, Carneiro MO, Hartl C, Poplin R, Del Angel G, Levy-Moonshine A (2013). From fastq data to high confidence variant calls: the genome analysis toolkit best practices pipeline. Curr Protoc Bioinform.

[13] Zhou G, Zhou M, Zeng F, Zhang N, Sun Y, Qiao Z (2022). Performance characterization of PCR-free whole genome sequencing for clinical diagnosis. Medicine (Baltimore).

[14] Thiffault I, Farrow E, Zellmer L, Berrios C, Miller N, Gibson M (2019). Clinical genome sequencing in an unbiased pediatric cohort. Genet Med.

[15] Chen S, Zhou Y, Chen Y, Gu J (2018). fastp: an ultra-fast all-in-one FASTQ preprocessor. Bioinformatics.

[16] Li H, Durbin R (2009). Fast and accurate short read alignment with burrows-wheeler transform. Bioinformatics.

[17] Rehm HL, Bale SJ, Bayrak-Toydemir P, Berg JS, Brown KK, Deignan JL (2013). ACMG clinical laboratory standards for next-generation sequencing. Genet Med.

[18] Cibulskis K, Lawrence MS, Carter SL, Sivachenko A, Jaffe D, Sougnez C (2013). Sensitive detection of somatic point mutations in impure and heterogeneous cancer samples. Nat Biotechnol.

[19] Abyzov A, Urban AE, Snyder M, Gerstein M (2011). CNVnator: an approach to discover, genotype, and characterize typical and atypical CNVs from family and population genome sequencing. Genome Res.

[20] Plagnol V, Curtis J, Epstein M, Mok KY, Stebbings E, Grigoriadou S (2012). A robust model for read count data in exome sequencing experiments and implications for copy number variant calling. Bioinformatics.

[21] Layer RM, Chiang C, Quinlan AR, Hall IM (2014). LUMPY: a probabilistic framework for structural variant discovery. Genome Biol.

[22] Shang X, Peng Z, Ye Y, Zhang X, Chen Y, Zhu B, Cai W, Chen S, Cai R, Guo X, Zhang C (2017). Rapid targeted next-generation sequencing platform for molecular screening and clinical genotyping in subjects with hemoglobinopathies. EBioMedicine.

[23] Purcell S, Neale B, Todd-Brown K, Thomas L, Ferreira MA, Bender D (2007). PLINK: a tool set for whole-genome association and population-based linkage analyses. Am J Hum Genet.

[24] Dolzhenko E, Deshpande V, Schlesinger F, Krusche P, Petrovski R, Chen S (2019). ExpansionHunter: a sequence-graph-based tool to analyze variation in short tandem repeat regions. Bioinformatics.

[25] Truong DT, Tett A, Pasolli E, Huttenhower C, Segata N (2017). Microbial strain-level population structure and genetic diversity from metagenomes. Genome Res.

[26] Wood DE, Lu J, Langmead B (2019). Improved metagenomic analysis with Kraken 2. Genome Biol.

[27] Lu J, Breitwieser FP, Thielen P, Salzberg SL (2017). Bracken: estimating species abundance in metagenomics data. PeerJ Comput Sci.

[28] Xiang J, Peng J, Baxter S, Peng Z (2020). AutoPVS1: an automatic classification tool for PVS1 interpretation of null variants. Hum Mutat.

[29] Fan C, Wang Z, Sun Y, Sun J, Liu X, Kang L (2021). AutoCNV: a semiautomatic CNV interpretation system based on the 2019 ACMG/ClinGen technical standards for CNVs. BMC Genomics.

[30] Jaganathan K, Kyriazopoulou Panagiotopoulou S, McRae JF, Darbandi SF, Knowles D, Li YI (2019). Predicting splicing from primary sequence with deep learning. Cell.

[31] Sherry ST, Ward MH, Kholodov M, Baker J, Phan L, Smigielski EM (2001). dbSNP: the NCBI database of genetic variation. Nucleic Acids Res.

[32] Landrum MJ, Lee JM, Benson M, Brown G, Chao C, Chitipiralla S (2016). ClinVar: public archive of interpretations of clinically relevant variants. Nucleic Acids Res.

[33] Cooper DN, Krawczak M (1996). Human gene mutation database. Hum Genet.

[34] Hamosh A, Scott AF, Amberger JS, Bocchini CA, McKusick VA (2005). Online mendelian inheritance in man (OMIM), a knowledgebase of human genes and genetic disorders. Nucleic Acids Res.

[35] Pavan S, Rommel K, Mateo Marquina ME, Hohn S, Lanneau V, Rath A (2017). Clinical practice guidelines for rare diseases: the orphanet database. PLoS ONE.

[36] Monaghan TF, Rahman SN, Agudelo CW, Wein AJ, Lazar JM, Everaert K (2021). Foundational statistical principles in medical research: sensitivity, specificity, positive predictive value, and negative predictive value. Medicina (Kaunas).

[37] Jian X, Boerwinkle E, Liu X (2014). In silico prediction of splice-altering single nucleotide variants in the human genome. Nucleic Acids Res.

[38] Leman R, Gaildrat P, Le Gac G, Ka C, Fichou Y, Audrezet MP (2018). Novel diagnostic tool for prediction of variant spliceogenicity derived from a set of 395 combined in silico/in vitro studies: an international collaborative effort. Nucleic Acids Res.

[39] Yeo G, Burge CB (2004). Maximum entropy modeling of short sequence motifs with applications to RNA splicing signals. J Comput Biol.

[40] Wilson GW, Derouet M, Darling GE, Yeung JC (2021). scSNV: accurate dscRNA-seq SNV co-expression analysis using duplicate tag collapsing. Genome Biol.

[41] Cheng J, Nguyen TYD, Cygan KJ, Celik MH, Fairbrother WG, Avsec Z (2019). MMSplice: modular modeling improves the predictions of genetic variant effects on splicing. Genome Biol.

[42] Zhang L, Bai W, Yuan N, Du Z (2019). Comprehensively benchmarking applications for detecting copy number variation. PLoS Comput Biol.

[43] Chen K, Wallis JW, McLellan MD, Larson DE, Kalicki JM, Pohl CS (2009). Break dancer: an algorithm for high-resolution mapping of genomic structural variation. Nat Methods.

[44] Rausch T, Zichner T, Schlattl A, Stutz AM, Benes V, Korbel JO (2012). DELLY: structural variant discovery by integrated paired-end and split-read analysis. Bioinformatics.

[45] Zhao S, Xiang J, Fan C, Shang X, Zhang X, Chen Y, Zhu B, Cai W, Chen S, Cai R, Guo X (2019). Pilot study of expanded carrier screening for 11 recessive diseases in China: results from 10,476 ethnically diverse couples. European J Human Genetics.

[46] Ibanez K, Polke J, Hagelstrom RT, Dolzhenko E, Pasko D, Thomas ERA (2022). Whole genome sequencing for the diagnosis of neurological repeat expansion disorders in the UK: a retrospective diagnostic accuracy and prospective clinical validation study. Lancet Neurol.

[47] Zhao S, Wang W, Wang Y, Han R, Fan C, Ni P (2021). NGS-based spinal muscular atrophy carrier screening of 10,585 diverse couples in China: a pan-ethnic study. Eur J Hum Genet.

[48] Prior TW, Professional P, Guidelines C (2008). Carrier screening for spinal muscular atrophy. Genet Med.

[49] Jegathisawaran J, Tsiplova K, Hayeems R, Ungar WJ (2020). Determining accurate costs for genomic sequencing technologies-a necessary prerequisite. J Community Genet.

[50] Smeets DF (2004). Historical prospective of human cytogenetics: from microscope to microarray. Clin Biochem.

[51] Guo X, Chen F, Gao F, Li L, Liu K, You L, Hua C, Yang F, Liu W, Peng C, Wang L, Yang X, Zhou F, Tong J, Cai J, Li Z, Wan B, Zhang L, Yang T, Zhang M, Yang L, Yang Y, Zeng W, Wang B, Wei X, Xu X (2020). CNSA: a data repository for archiving omics data. Database (Oxford).

[52] Chen FZ, You LJ, Yang F, Wang LN, Guo XQ, Gao F (2020). CNGBdb: China national genebank database. Yi Chuan.

