# Supplementary material for: Test development, optimization and validation of a WGS pipeline for genetic disorders
Source: BMC Med Genomics. 2023 Apr 5;16:74. doi: 10.1186/s12920-023-01495-x (PMC10077614; doi:10.1186/s12920-023-01495-x)
Supplement: Supplementary file 1 — Additional file 1. Supplementary Methods, Figures and Tables. [file 12920_2023_1495_MOESM1_ESM.docx]

Supplementary Material

# Supplementary Methods

**Determination of optimal window size (each window contains a specific number of SNPs) for detecting AOHs**

1. Building the training set and test set

To determine the optimal window size, we included 101 known positive AOH regions (length ≥ 3M) from 12 clinical samples (data not shown). These AOH regions were then divided into a training set and a test set (1:1).

1. Finding the optimal window size using the training set

Using the training set, different window sizes were set together with other software parameters of the Plink program. All combinations of different software parameters were traversed to choose an optimal combination. For each combination of parameters, the overlap between the detected AOH region and the known AOH region was calculated in the training set. Then each overlap was added together to get the total overlap between the detected AOH regions and the known AOH regions in the training set. Then we obtain the largest overlap with a certain combination. Under this combination, the window size was selected as the optimal window size (each window contains 20 SNPs).

1. Validation of the optimal window size using the test set

We further validated the optimal window size (each window contains 20 SNPs) using the test set. After calculation, a total overlap of 97.77% was obtained when using the optimal window size.

# Supplementary Figures and Tables

## 2.1 Supplementary Figures

**Supplementary Figure 1.** Comparison of 4 tools using 4,605 variants. We compared the detection sensitivity, specificity, accuracy, and prediction rate of 4 tools (SpliceAI, MaxEntScan, scSNV and MMSplice) for the prediction of splice-altering variants in the human genome. A total of 4,605 variants were used for comparison. These variants were derived from 2 published articles [1, 2] and the HGMD database (excluding training sets and overlaps among different sets), including 4,513 positive variants and 92 negative variants. As a result, the detection sensitivity, specificity, accuracy, and prediction rat e of SpliceAI were 96.80%, 85.87%, 96.58%, and 99.59% respectively. After comprehensively comparison, SpliceAI was implemented for the detection of splice variants in our pipeline.

F**igures for showing aneuploidies and triploidies (Supplementary Figure 2-7)**

Figure A is the distribution of the detected SNP ratio of the chromosome. The green solid line is the result of mixed Gaussian model fitted with 2 components. The r-square value represents the correlation between the fitted probability density and the actual probability density distribution of detected SNP ratio. As some chromosomes have less variations or greater fluctuation, the r-square of the fitted data may be relatively small (Supplementary Figure 4-5), which requires manual review. The orange dotted line is the clustering result of two peaks. A normal diploid has only one peak. Figure B is the distribution of the detected SNP ratio, each point in the figure represents a SNP. The X-axis shows the genomic coordinates of the chromosome, and the Y-axis shows the SNP ratio value. The two blue areas in figure B correspond to the two peaks of figure A. Figure C is the normalized depth, for example, a depth of 3 means 3 copies.

For example, if only one chromosome of a case has two peaks in figure A and the normalized depth in figure C is around 3, and the other chromosomes are normal, it is identified as a trisomy. If all chromosomes have two peaks in figure A and all chromosomes have a normalized depth of about 3 in figure C, it is identified as a triploidy.


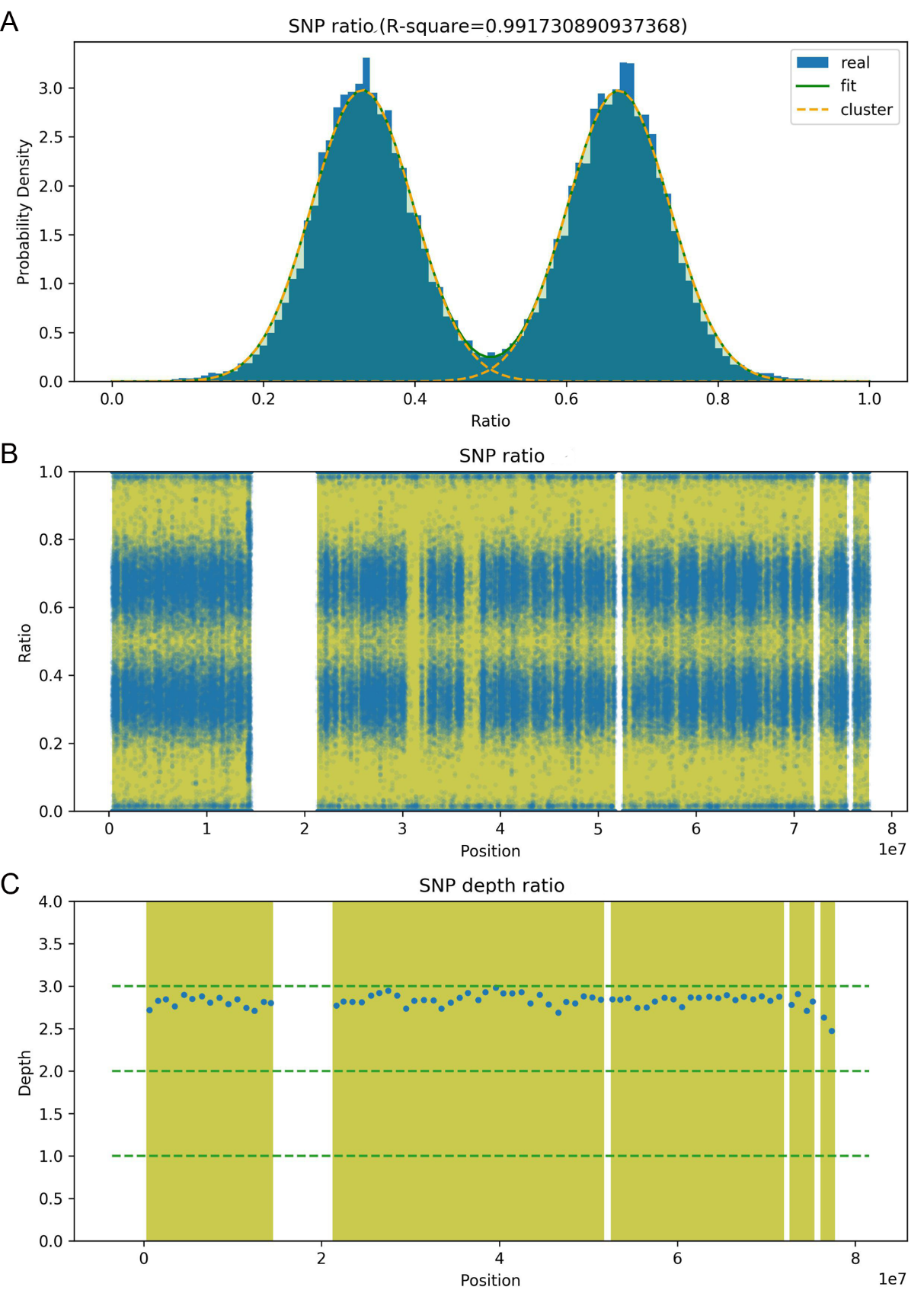


**Supplementary Figure 2.** WGS results for Case 9. A. Binomial distribution of SNP ratio for chromosome 18. B. SNP ratio distribution of chromosome 18. C. SNP depth ratio distribution of chromosome 18. Chromosome 18 has two peaks in figure A and the normalized depth in figure C is close to 3, and the other chromosomes are normal. One aneuploidy (seq[GRCh37] (18)x3) was identified in Case 9.

**
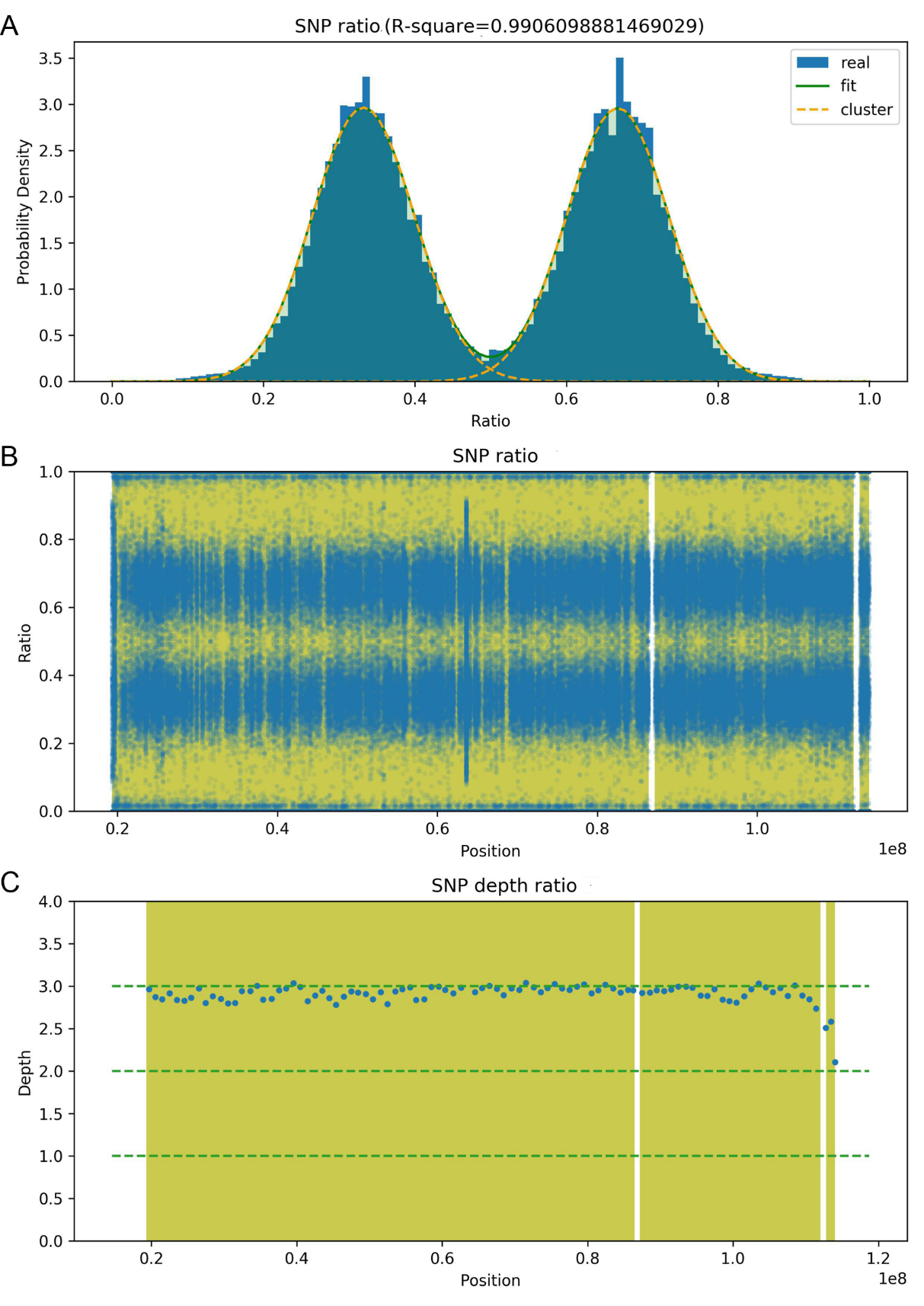
Supplementary Figure 3.** WGS results for Case 10. A. Binomial distribution of SNP ratio for chromosome 13. B. SNP ratio distribution of chromosome 13. C. SNP depth ratio distribution of chromosome 13. Chromosome 13 has two peaks in figure A and the normalized depth in figure C is around 3, and the other chromosomes are normal. One aneuploidy (seq[GRCh37] (13)x3) was identified in Case 10.

**
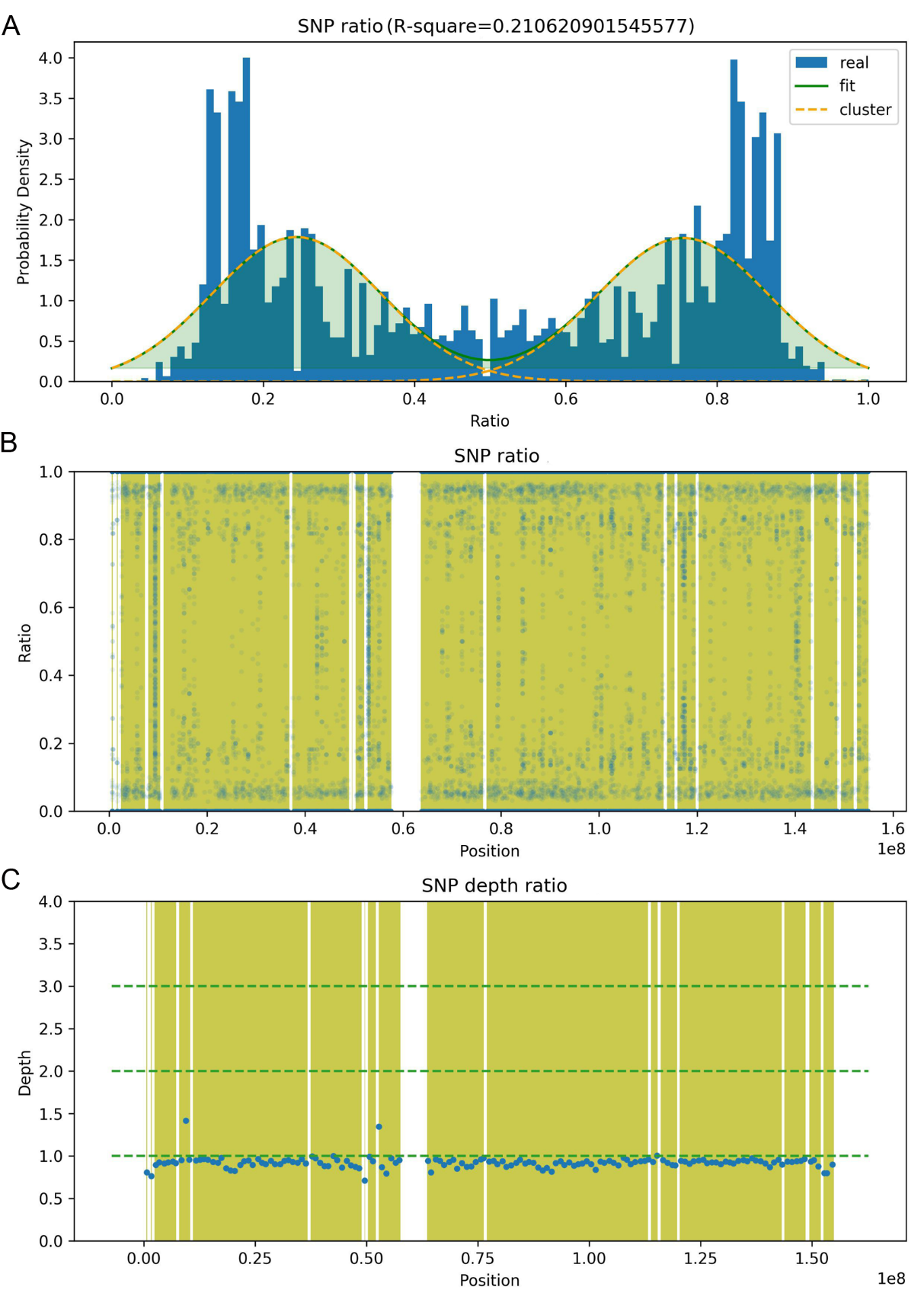
Supplementary Figure 4.** WGS results for Case 11. A. Binomial distribution of SNP ratio for chromosome X. B. SNP ratio distribution of chromosome X. C. SNP depth ratio distribution of chromosome X. Chromosome X has two peaks in figure A and the normalized depth in figure C is close to 1, and the other chromosomes are normal. One aneuploidy (seq[GRCh37] (X)x1) was identified in Case 11.

**
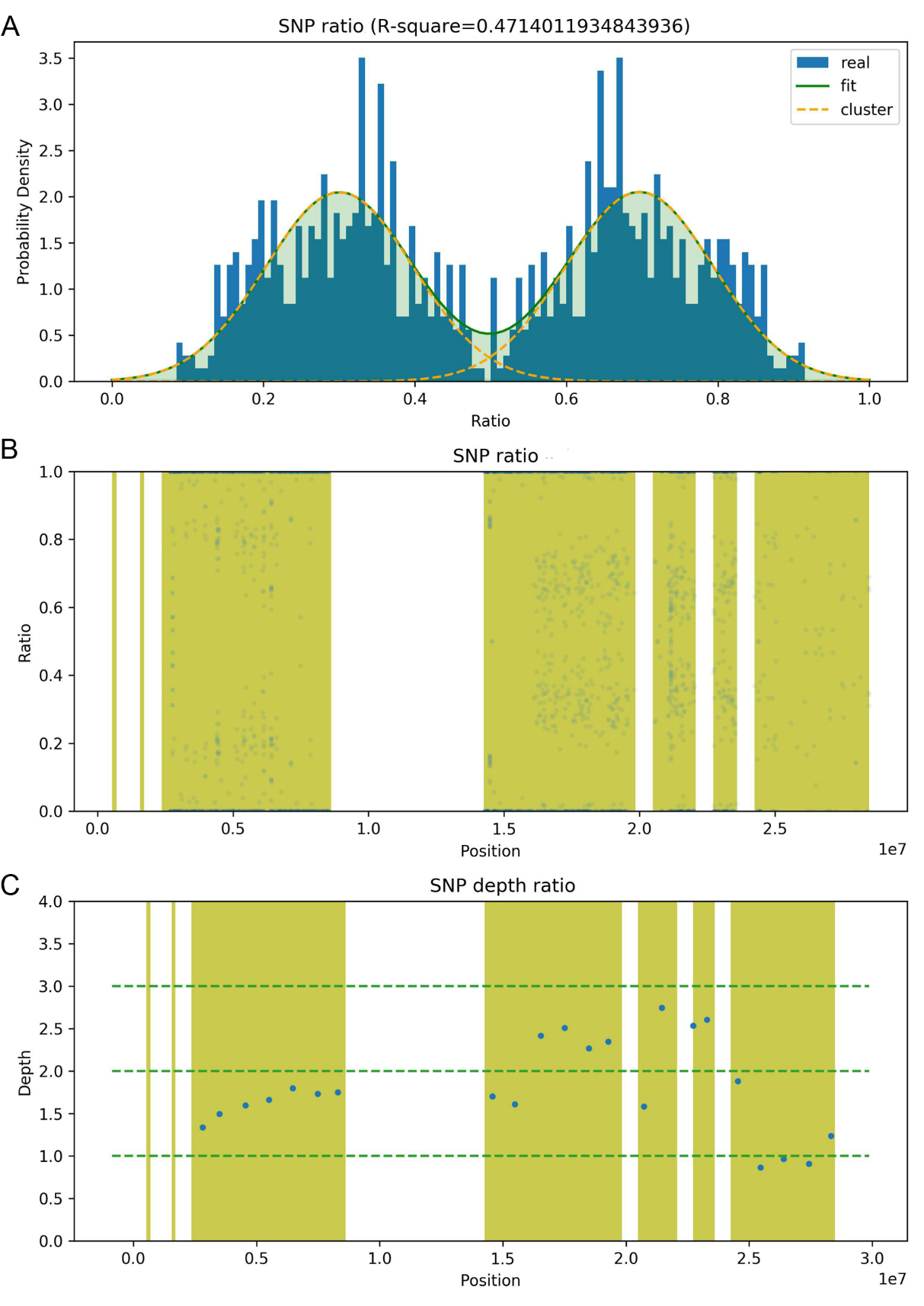
**

**Supplementary Figure 5.** WGS results for Case 12. A. Binomial distribution of SNP ratio for chromosome Y. B. SNP ratio distribution of chromosome Y. C. SNP depth ratio distribution of chromosome Y. Chromosome Y has two peaks in figure A and the normalized depth in figure C is around 2, and the other chromosomes are normal. One aneuploidy (seq[GRCh37] (X)x1,(Y)x2) was identified in Case 12.

**
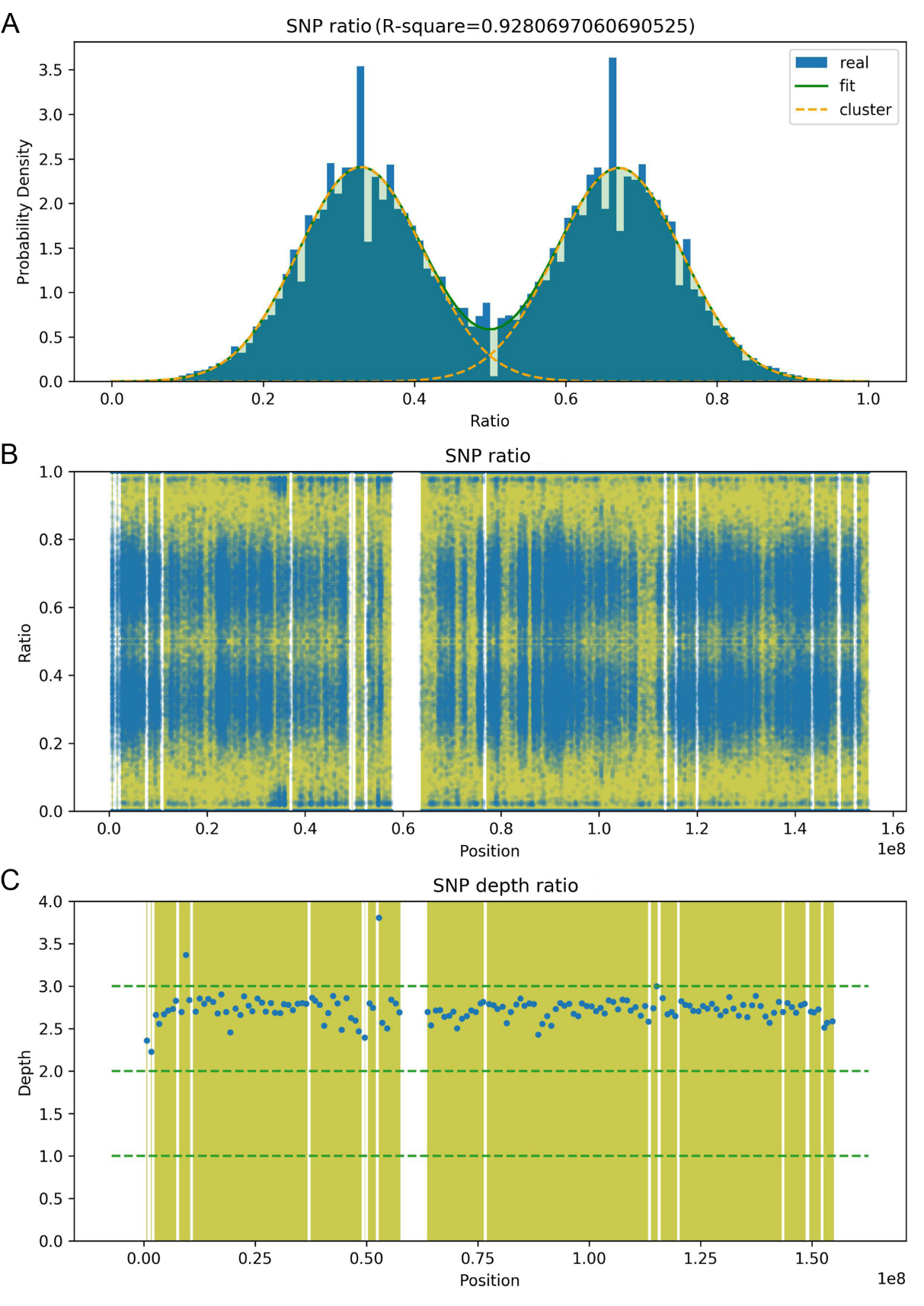
**

**Supplementary Figure 6.** WGS results for Case 13. A. Binomial distribution of SNP ratio for chromosome X. B. SNP ratio distribution of chromosome X. C. SNP depth ratio distribution of chromosome X. All chromosomes have two peaks in figure A and the normalized depth in figure C is around 3 (only figures for chromosome X are shown). One triploidy (seq[GRCh37] (X,1-22)x3) was identified in Case 13.

**
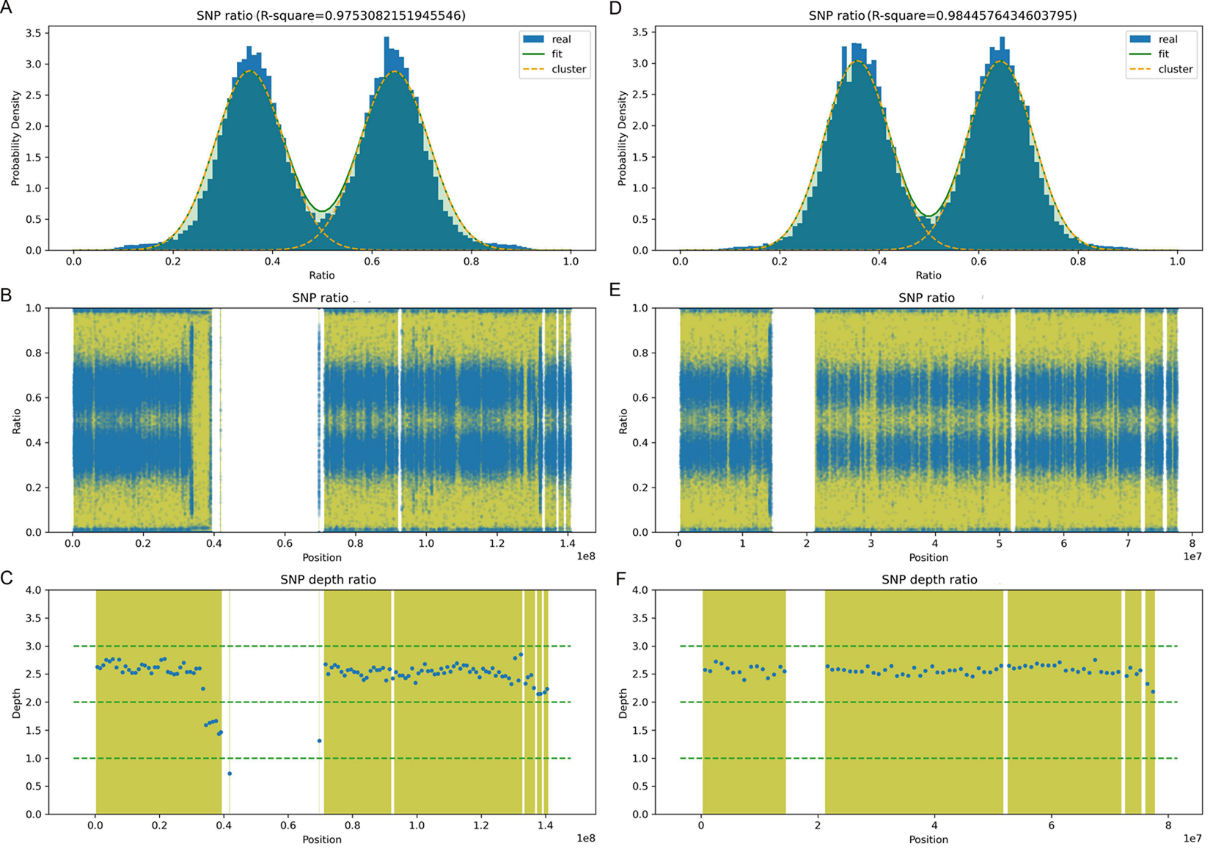
Supplementary Figure 7.** WGS results for NA12722. A. Binomial distribution of SNP ratio for chromosome 9. B. SNP ratio distribution of chromosome 9. C. SNP depth ratio distribution of chromosome 9. D. Binomial distribution of SNP ratio for chromosome 18. E. SNP ratio distribution of chromosome 18. F. SNP depth ratio distribution of chromosome 18. Both chromosome 9 and chromosome 18 have two peaks (figure A, figure D) and the normalized depth is close to 3 except for a deletion region of chromosome 9 (figure C, figure F), and the other chromosomes are normal. One aneuploidy and one deletion were identified on chromosome 9 and one aneuploidy was identified on chromosome 18 in NA12722 as following: seq[GRCh37] (9,18)x3 del(9)(p13.3p13.1) chr9:g.33416001_40991000del.

**Figures for showing balanced translocations (Supplementary Figure 8-12)**

Balanced translocations were visualized using the Integrative Genomics Viewer (IGV) [3]. IGV user guide (https://software.broadinstitute.org/software/igv/AlignmentData) provides detailed description on how to see these figures.


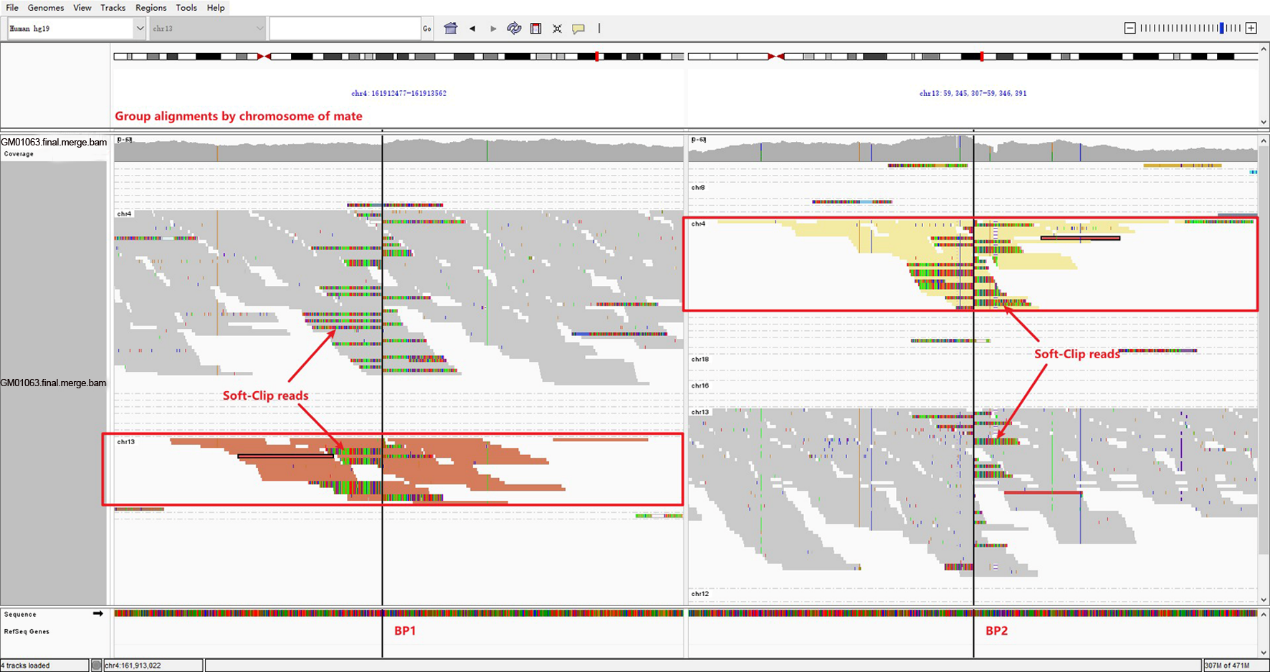


**Supplementary Figure 8.** WGS results for GM01063. One balanced translocation was identified in GM01063 as following: seq[GRCh37] t(4;13)(q32.2(161913247);q21.1(59345836)).


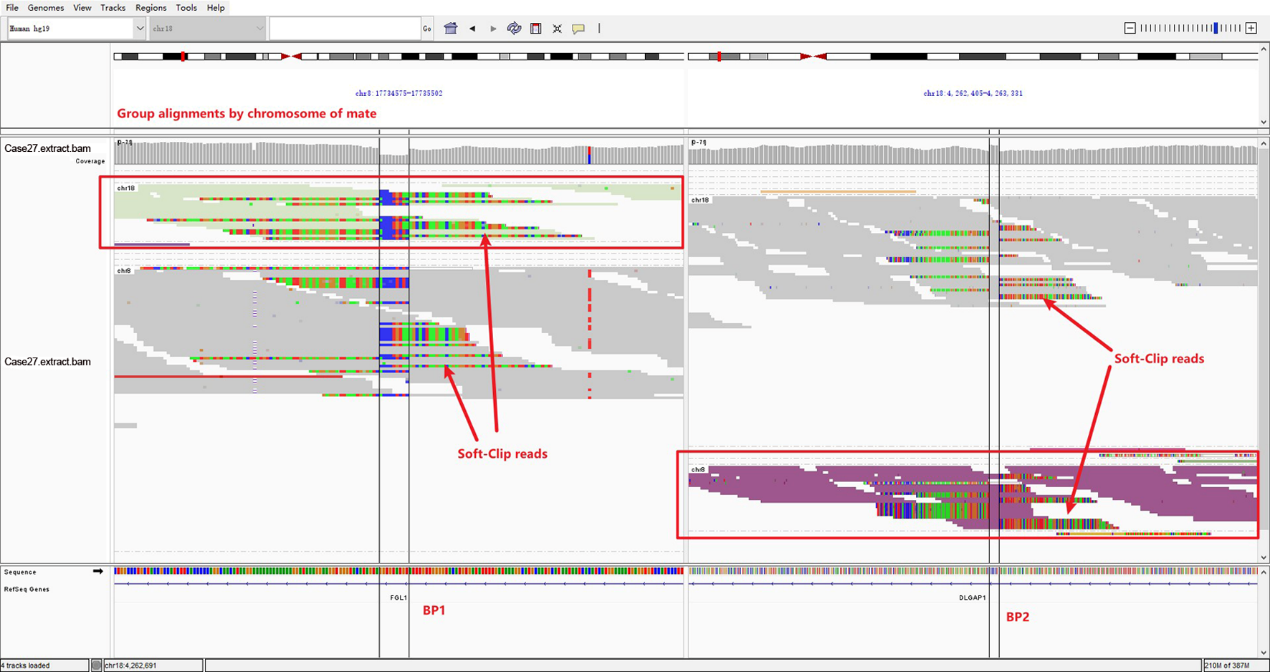


**Supplementary Figure 9.** WGS results for Case 27. One balanced translocation was identified in Case 27 as following: seq[GRCh37] t(8;18)(p22(17735160_17735170);p11.31(4262857_4262862)).


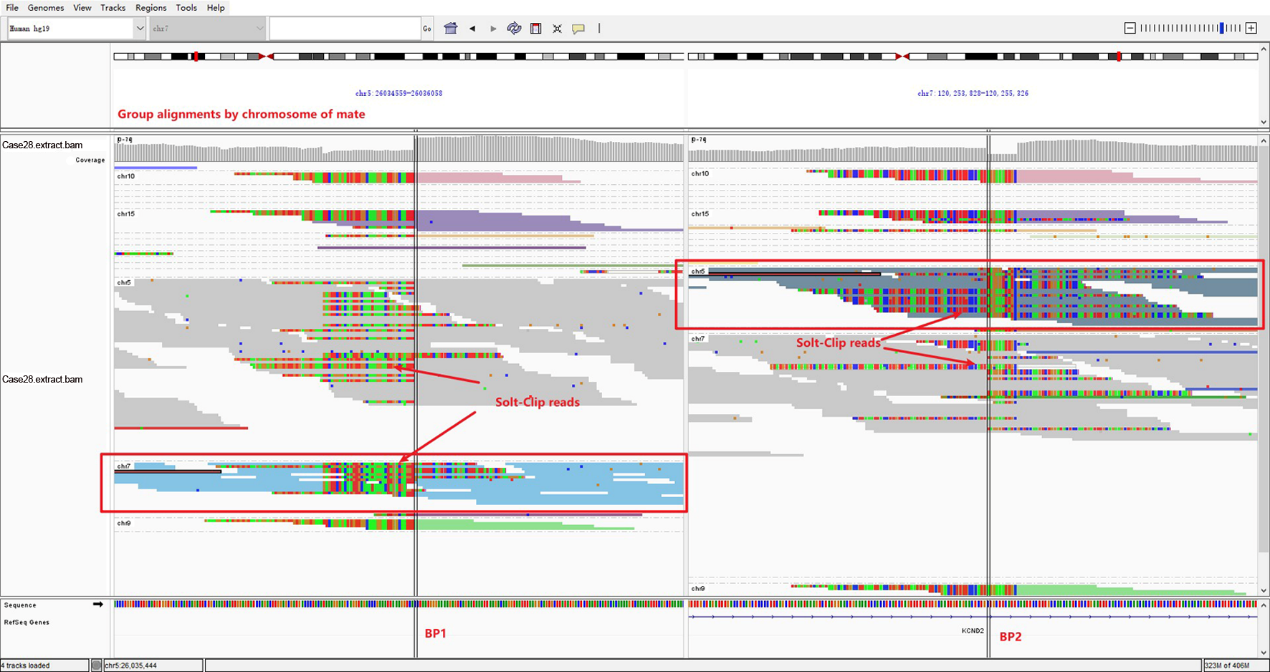


**Supplementary Figure 10.** WGS results for Case 28. One balanced translocation was identified in Case 28 as following: seq[GRCh37] t(5;7)(p14.1(26035448);q31.31(120254553)).


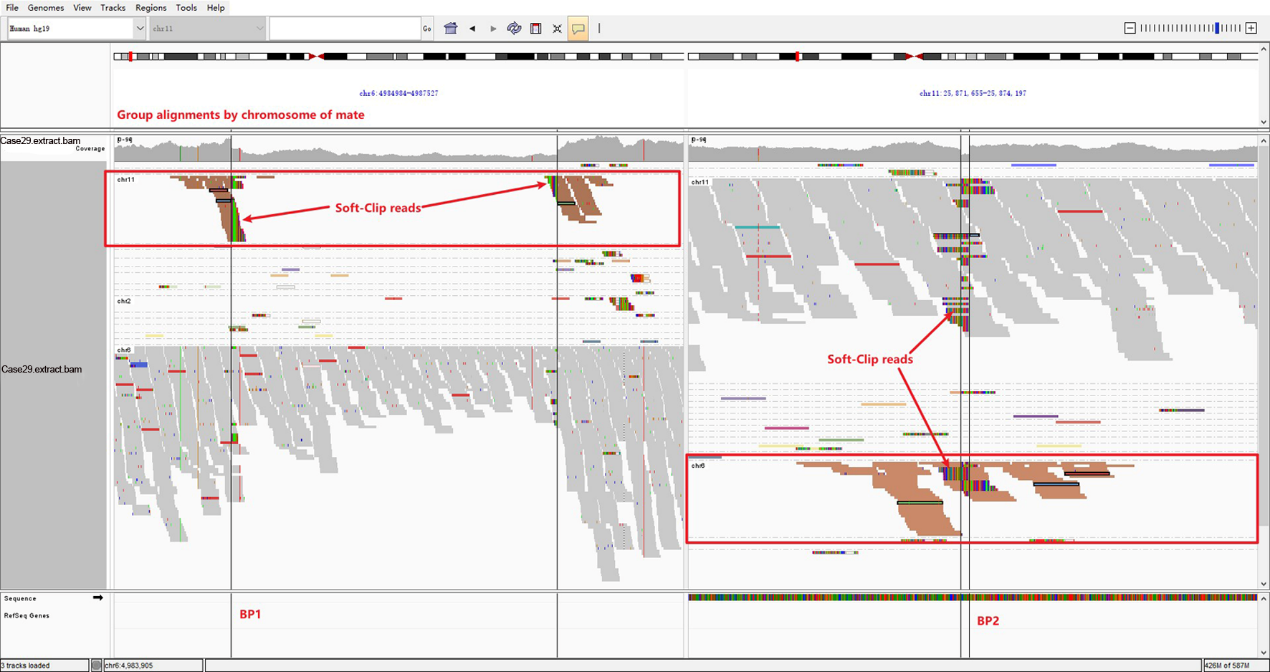


**Supplementary Figure 11.** WGS results for Case 29. One balanced translocation was identified in Case 29 as following: seq[GRCh37] t(6;11)(p25.1(4984417_4986257);p14.3(25872804_25872824)).


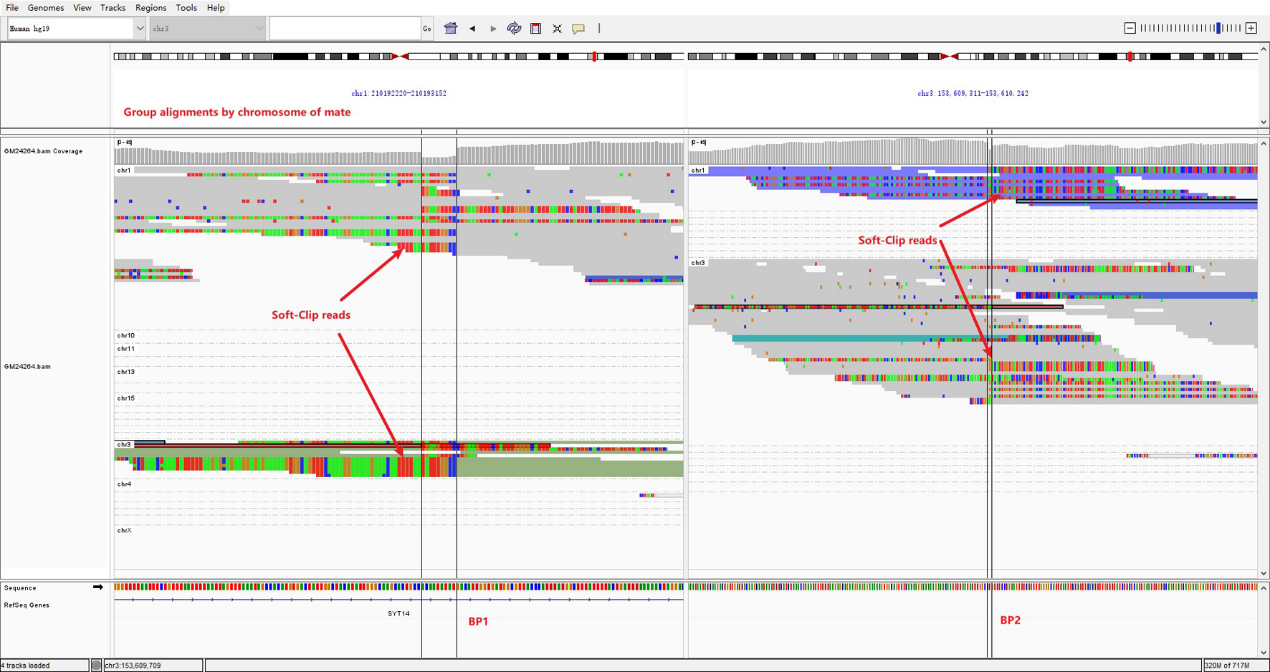


**Supplementary Figure 12.** WGS results for GM24264. One balanced translocation was identified in Case 29 as following: seq[GRCh37] t(1;3)(q32.1(210192903_210192913);q25.2(153609776_153609774))

**Figures for showing AOHs (Supplementary Figure 13-29)**

The X axis shows genomic coordinates of the chromosome, the Y axis shows the number of SNPs at each position. The light orange solid line represents all SNPs, the light blue solid line represents heterozygous SNPs. The light green region is the AOH region detected by WGS.


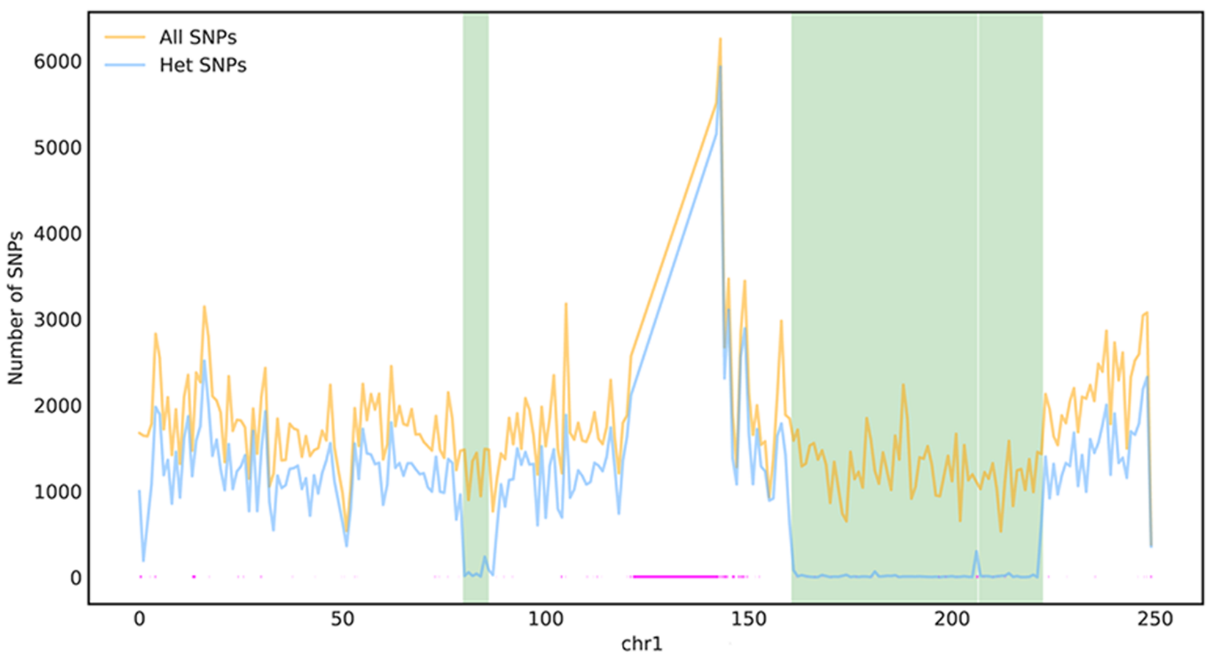
 **Supplementary Figure 13.** WGS results for chromosome 1 in NA00338. Three AOH regions highlighted in light green were identified on chromosome 1 in NA00338 as following: seq[GRCh37] hmz(1)(p31.1p22.2) chr1:g.79638609_85981487hmz; hmz(1)(q23.2q41) chr1:g.160474013_206309249hmz; hmz(1)(q23.2q41) chr1:g.206566766_222379937hmz.


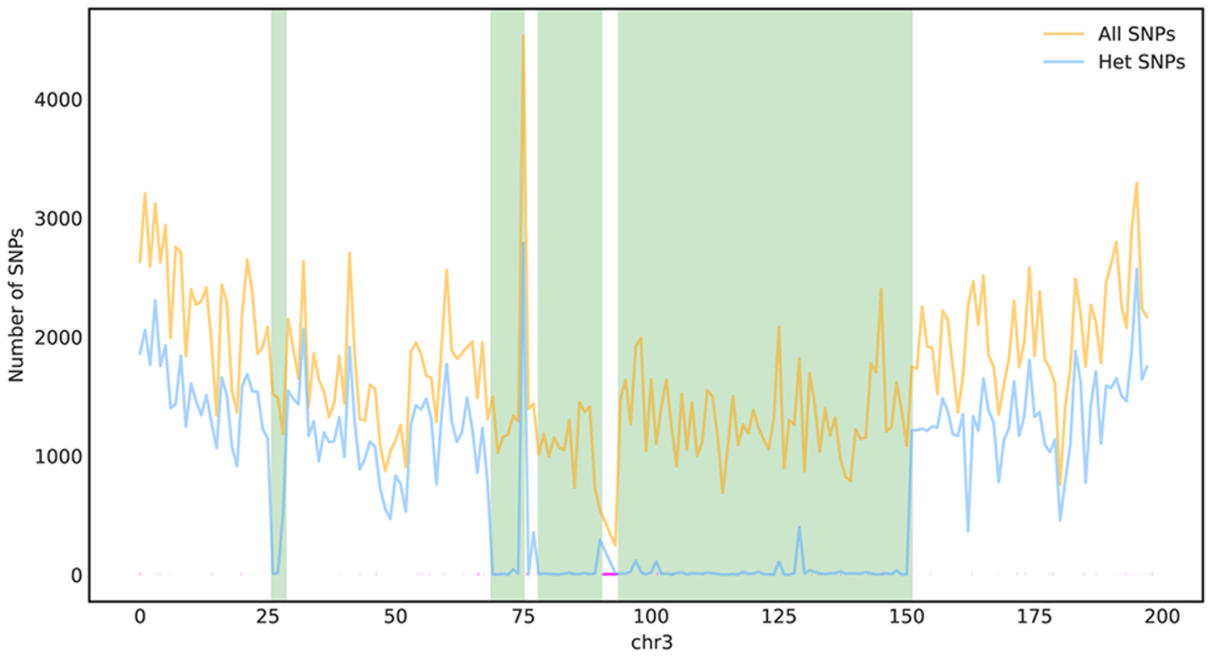


**Supplementary Figure 14.** WGS results for chromosome 3 in NA00338. Five AOH regions highlighted in light green were identified on chromosome 3 in NA00338 as following: seq[GRCh37] hmz(3)(p24.2p24.1) chr3:g.25643989_28680907hmz; hmz(3)(p14.1q25.1) chr3:g.68571007_75270810hmz; hmz(3)(p14.1q25.1) chr3:g.77835431_90450435hmz; hmz(3)(p14.1q25.1) chr3:g.93509006_129799485hmz; hmz(3)(p14.1q25.1) chr3:g.129809474_151171901hmz.


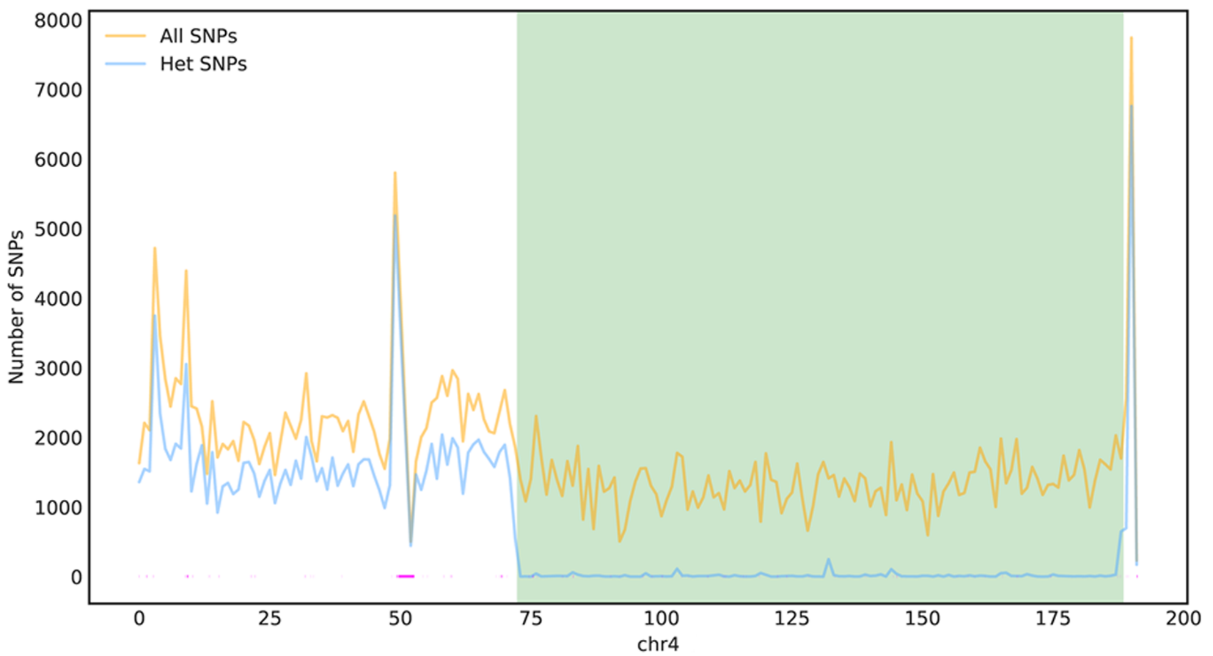


**Supplementary Figure 15.** WGS results for chromosome 4 in NA00338. Two AOH regions highlighted in light green were identified on chromosome 4 in NA00338 as following: seq[GRCh37] hmz(4)(q13.3q35.2) chr4:g.72346616_132653117hmz; hmz(4)(q13.3q35.2) chr4:g.132671346_188485278hmz.


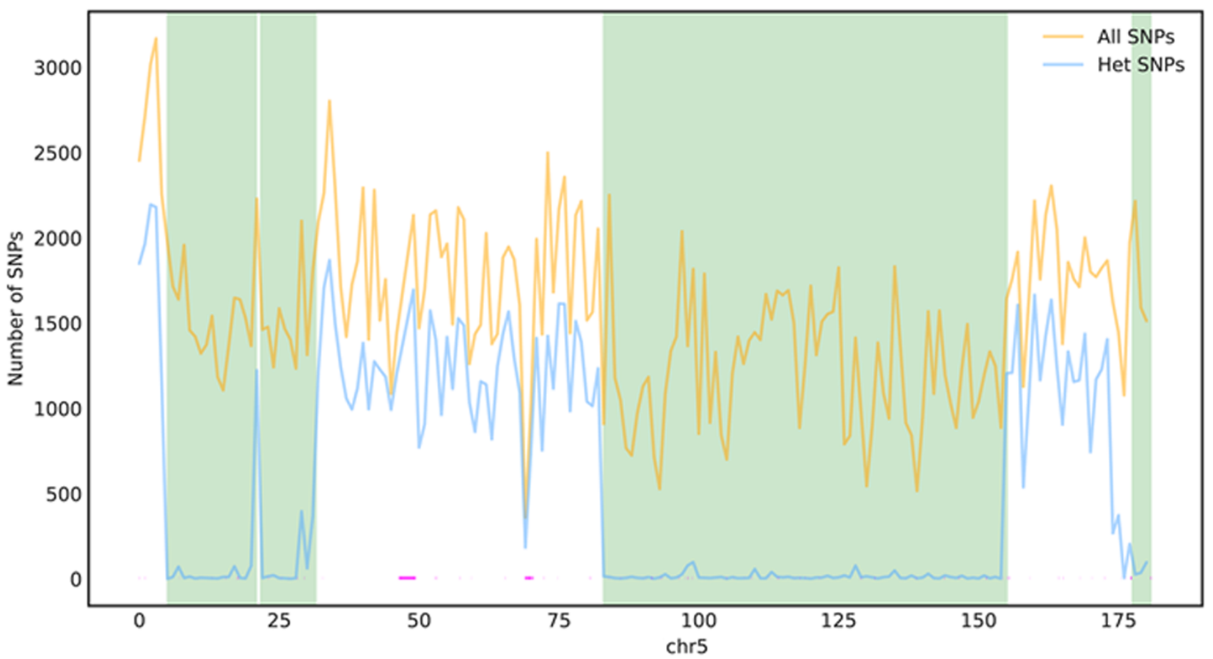


**Supplementary Figure 16.** WGS results for chromosome 5 in NA00338. Five AOH regions highlighted in light green were identified on chromosome 5 in NA00338 as following: seq[GRCh37] hmz(5)(p15.32p13.3) chr5:g.4913484_21082817hmz; hmz(5)(p15.32p13.3) chr5:g.21572713_29425215hmz; hmz(5)(p15.32p13.3) chr5:g.29434245_31702283hmz; hmz(5)(q14.3q33.2) chr5:g.82852908_155197081hmz; hmz(5)(q35.2q35.3) chr5:g.177348614_180903190hmz.


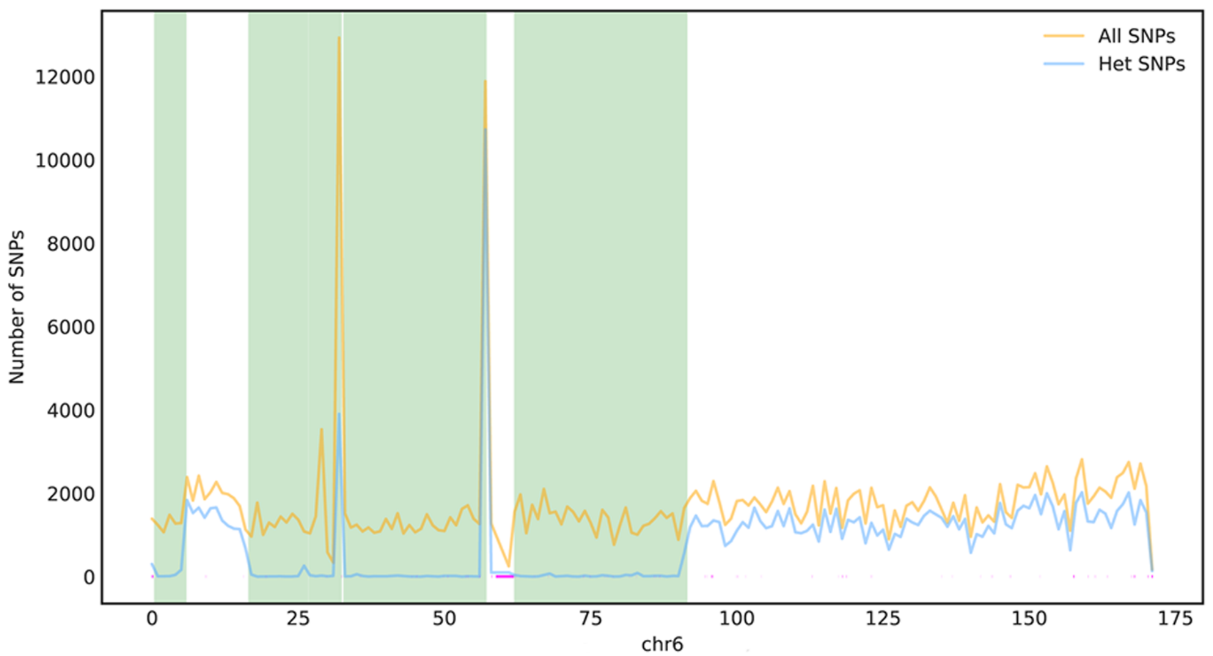
 **Supplementary Figure 17.** WGS results for chromosome 6 in NA00338. Five AOH regions highlighted in light green were identified on chromosome 6 in NA00338 as following: seq[GRCh37] hmz(6)(p25.3p25.1) chr6:g.360038_5910305hmz; hmz(6)(p22.3q15) chr6:g.16475066_26727284hmz; hmz(6)(p22.3q15) chr6:g.26775039_32440405hmz; hmz(6)(p22.3q15) chr6:g.32728902_57206220hmz; hmz(6)(p22.3q15) chr6:g.61920506_91544177hmz.


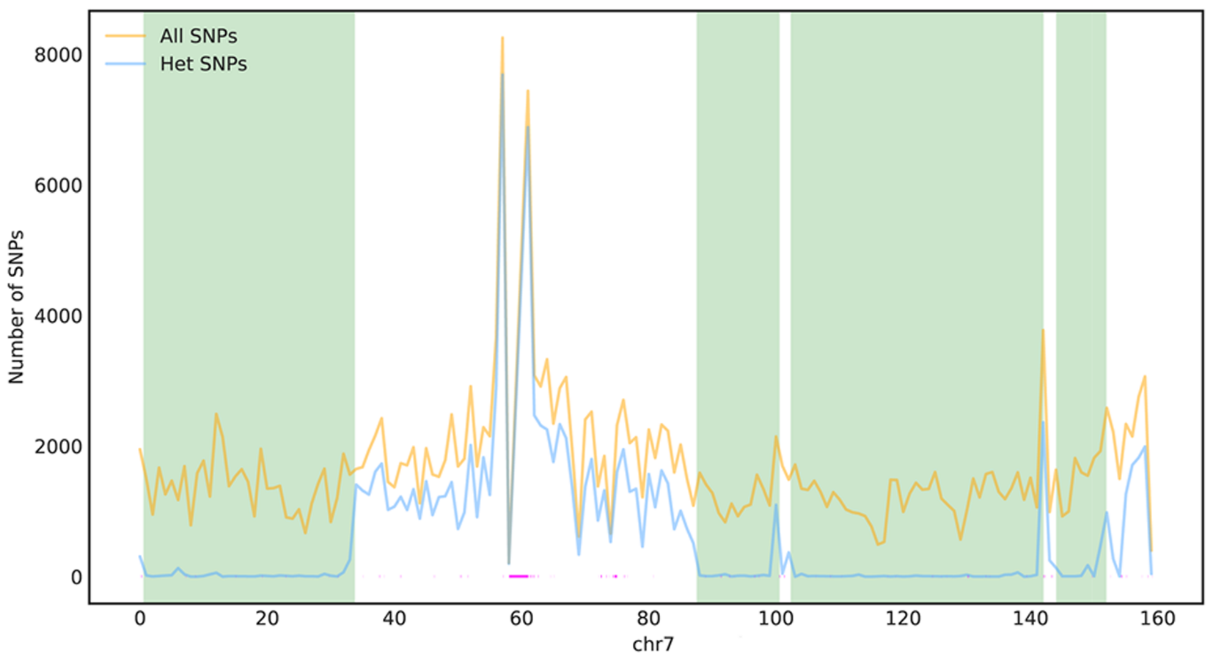


**Supplementary Figure 18.** WGS results for chromosome 7 in NA00338. Five AOH regions highlighted in light green were identified on chromosome 7 in NA00338 as following: seq[GRCh37] hmz(7)(p22.3p14.3) chr7:g.550944_33777808hmz; hmz(7)(q21.12q36.3) chr7:g.87533090_100546815hmz; hmz(7)(q21.12q36.3) chr7:g.102314531_142047342hmz; hmz(7)(q21.12q36.3) chr7:g.144048199_149725582hmz; hmz(7)(q21.12q36.3) chr7:g.149744605_151935567hmz.


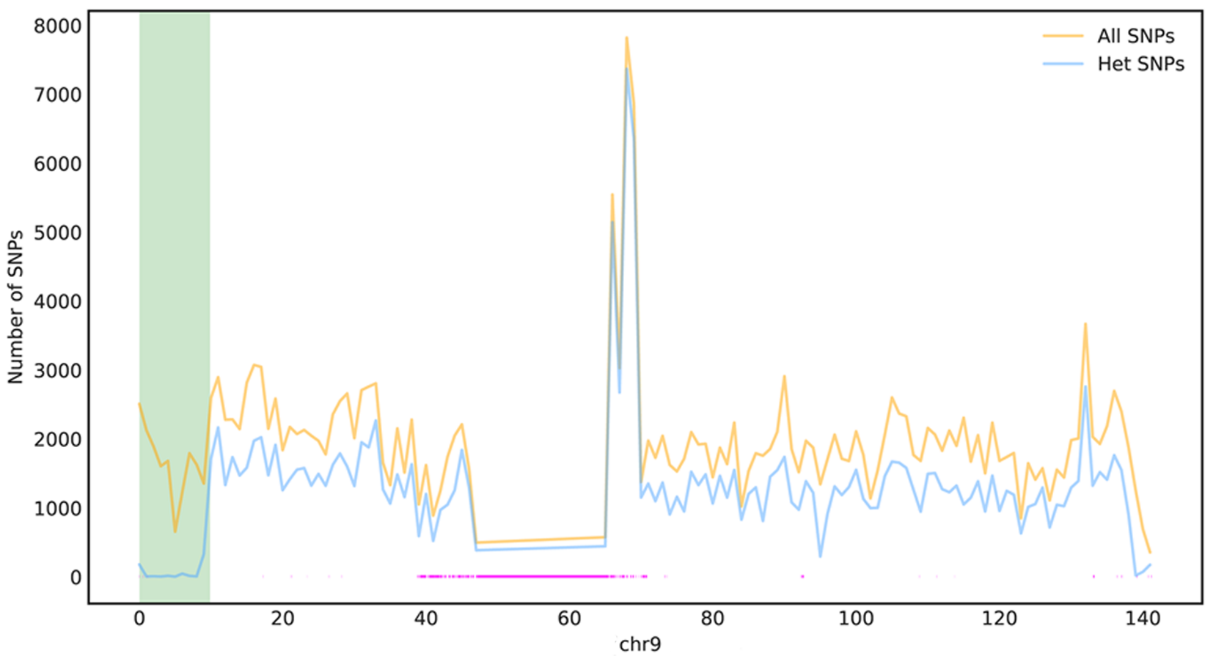
**Supplementary Figure 19.** WGS results for chromosome 9 in NA00338. One AOH region highlighted in light green was identified on chromosome 9 in NA00338 as following: seq[GRCh37] hmz(9)(p24.3p23) chr9:g.31589_9866177hmz.


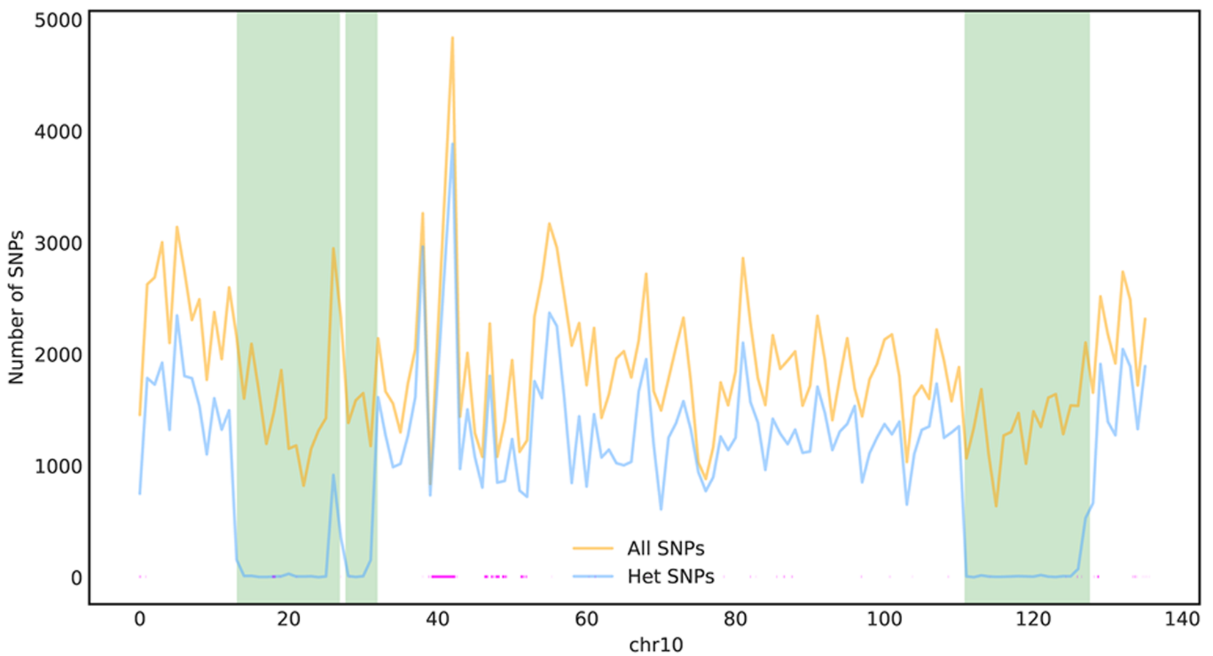


**Supplementary Figure 20.** WGS results for chromosome 10 in NA00338. Three AOH regions highlighted in light green were identified on chromosome 10 in NA00338 as following: seq[GRCh37] hmz(10)(p13p11.22) chr10:g.13027881_26860043hmz; hmz(10)(q25.1q26.2) chr10:g.27621971_31916276hmz; hmz(10)(q25.1q26.2) chr10:g.110757671_127577066hmz.


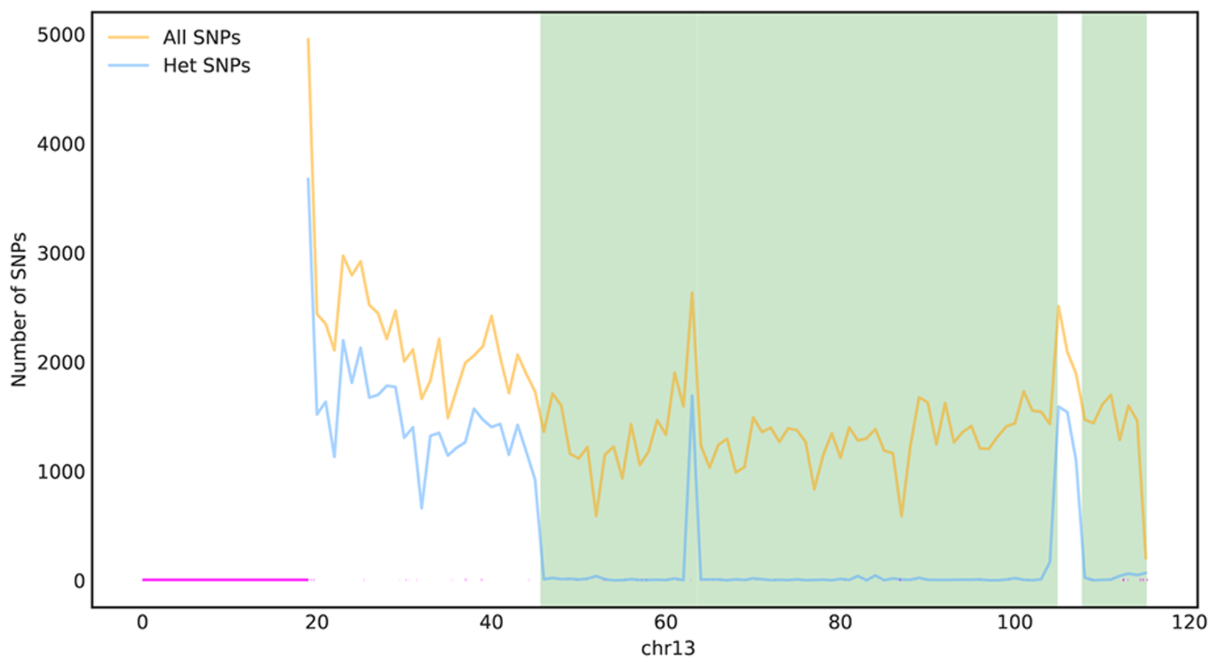


**Supplementary Figure 21.** WGS results for chromosome 13 in NA00338. Three AOH regions highlighted in light green were identified on chromosome 13 in NA00338 as following: seq[GRCh37] hmz(13)(q14.12q33.2) chr13:g.45607568_63603241hmz; hmz(13)(q33.3q34) chr13:g.63648556_104875972hmz; hmz(13)(q33.3q34) chr13:g.107654799_115109706hmz.


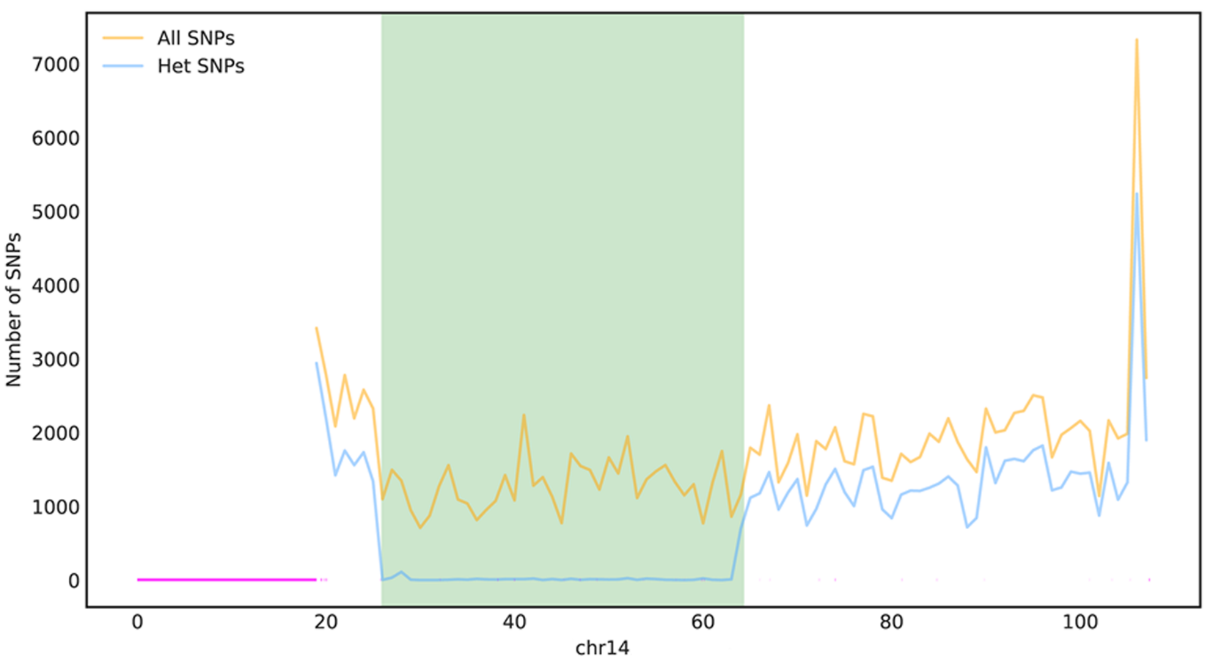


**Supplementary Figure 22.** WGS results for chromosome 14 in NA00338. One AOH region highlighted in light green was identified on chromosome 14 in NA00338 as following: seq[GRCh37] hmz(14)(q12q23.2) chr14:g.25888146_64349064hmz.


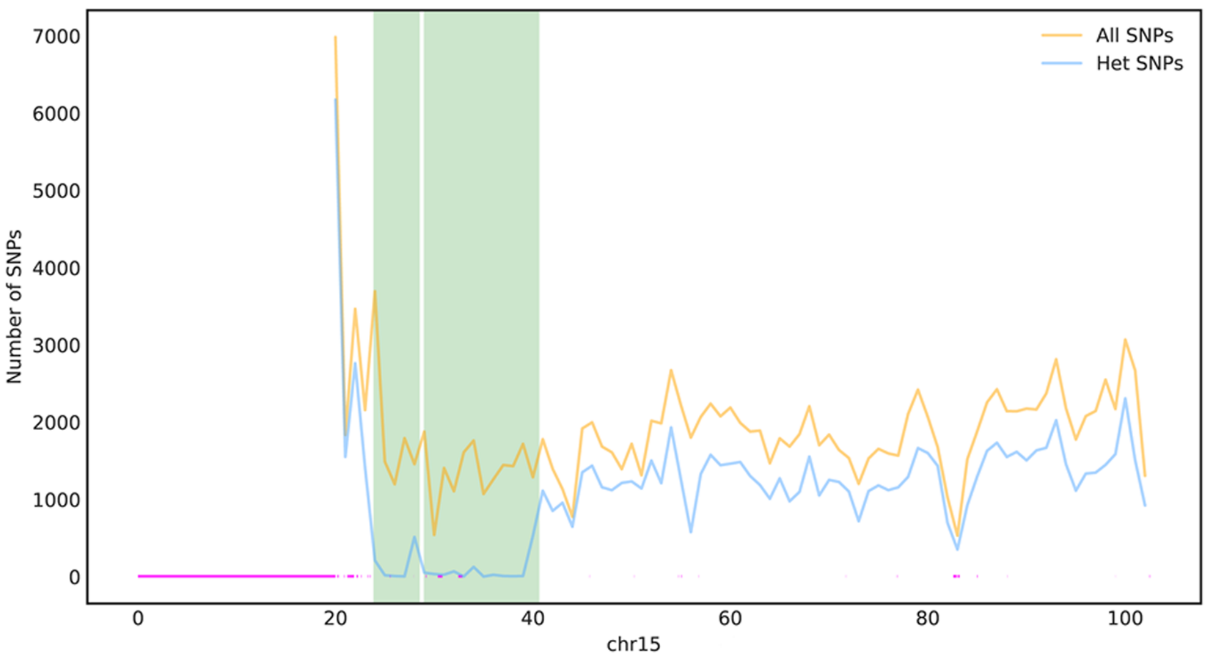
 **Supplementary Figure 23.** WGS results for chromosome 15 in NA00338. Two AOH regions highlighted in light green were identified on chromosome 15 in NA00338 as following: seq[GRCh37] hmz(15)(q11.2q15.1) chr15:g.23851100_28538769hmz; hmz(15)(q11.2q15.1) chr15:g.28957120_40641447hmz.


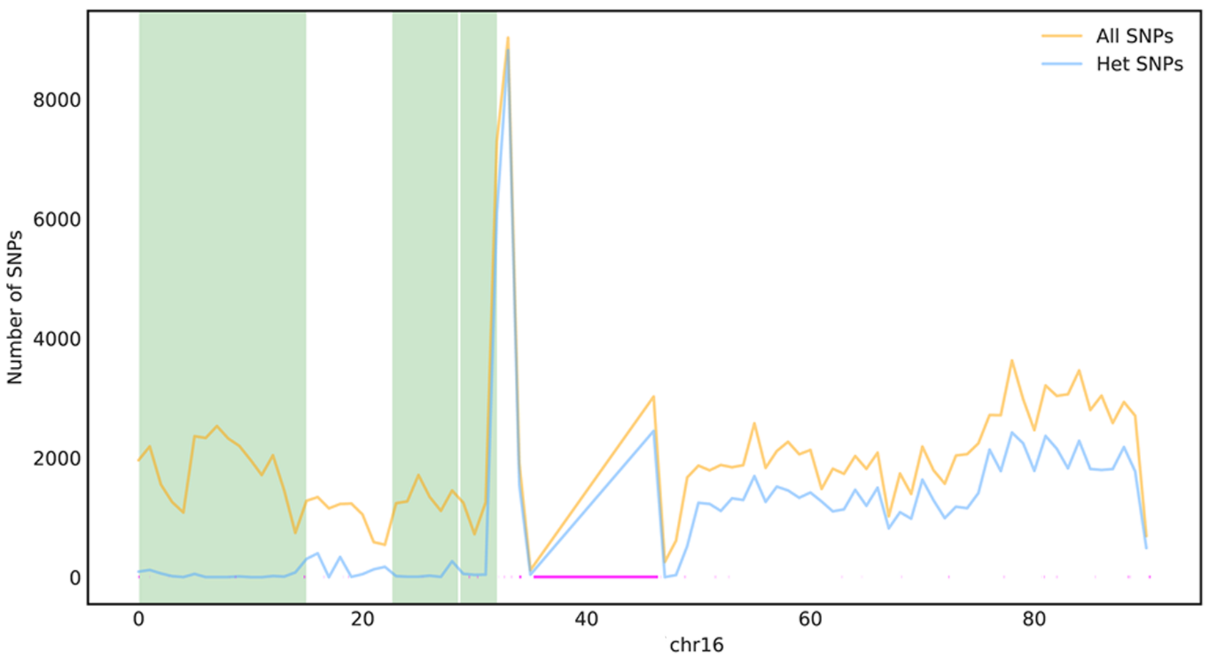


**Supplementary Figure 24.** WGS results for chromosome 16 in NA00338. Three AOH regions highlighted in light green were identified on chromosome 16 in NA00338 as following: seq[GRCh37] hmz(16)(p13.3q12.1) chr16:g.60291_14972915hmz; hmz(16)(p13.3q12.1) chr16:g.22671215_28531357hmz; hmz(16)(p13.3q12.1) chr16:g.28737086_32006758hmz.


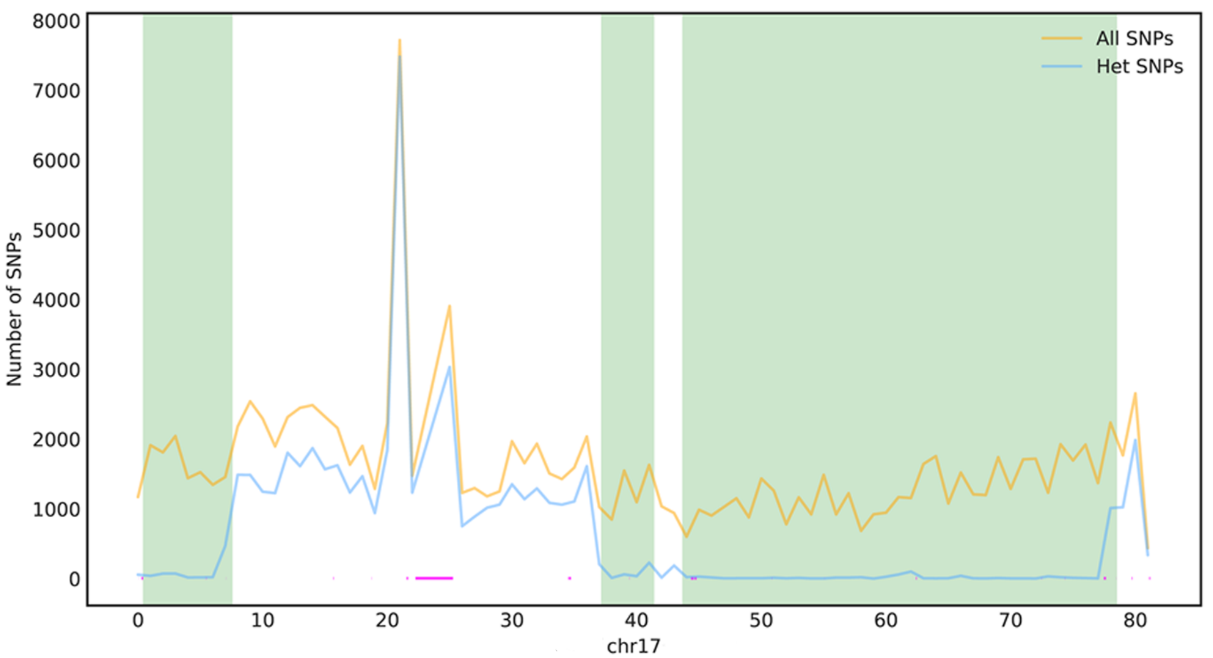


**Supplementary Figure 25.** WGS results for chromosome 17 in NA00338. Three AOH regions highlighted in light green were identified on chromosome 17 in NA00338 as following: seq[GRCh37] hmz(17)(p13.3p13.1) chr17:g.396660_7584400hmz; hmz(17)(q12q25.3) chr17:g.37145222_41408858hmz; hmz(17)(q12q25.3) chr17:g.43662883_78528340hmz.


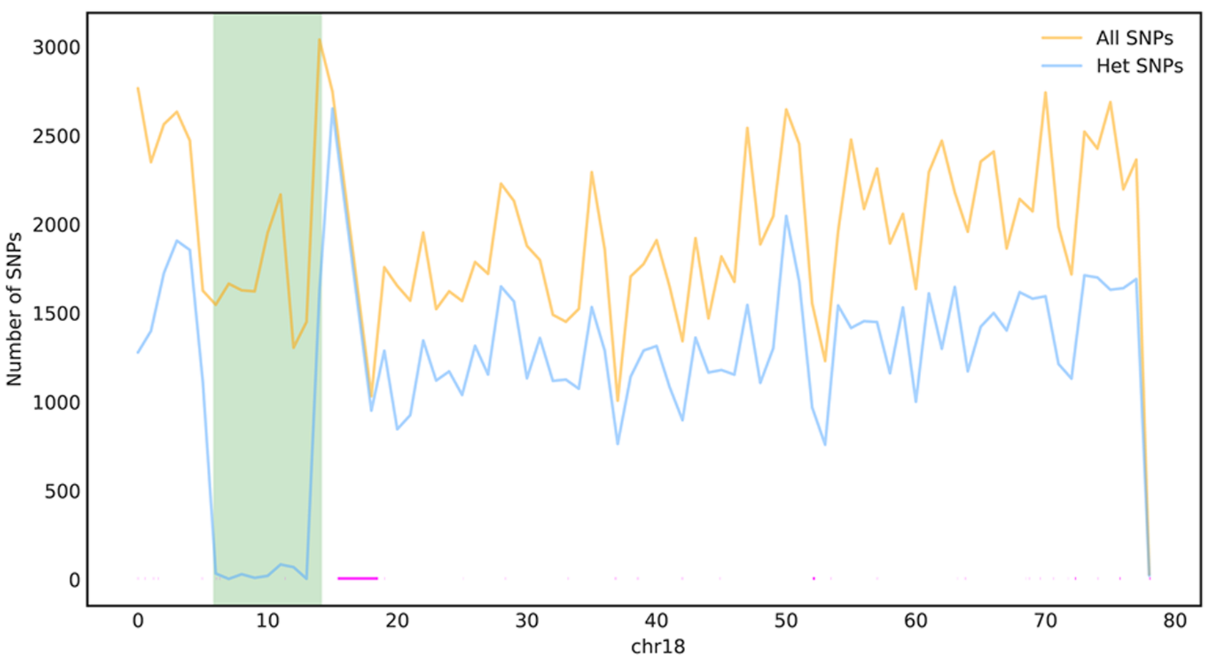


**Supplementary Figure 26.** WGS results for chromosome 18 in NA00338. One AOH region highlighted in light green was identified on chromosome 18 in NA00338 as following: seq[GRCh37] hmz(18)(p11.31p11.21) chr18:g.5812234_14182114hmz.


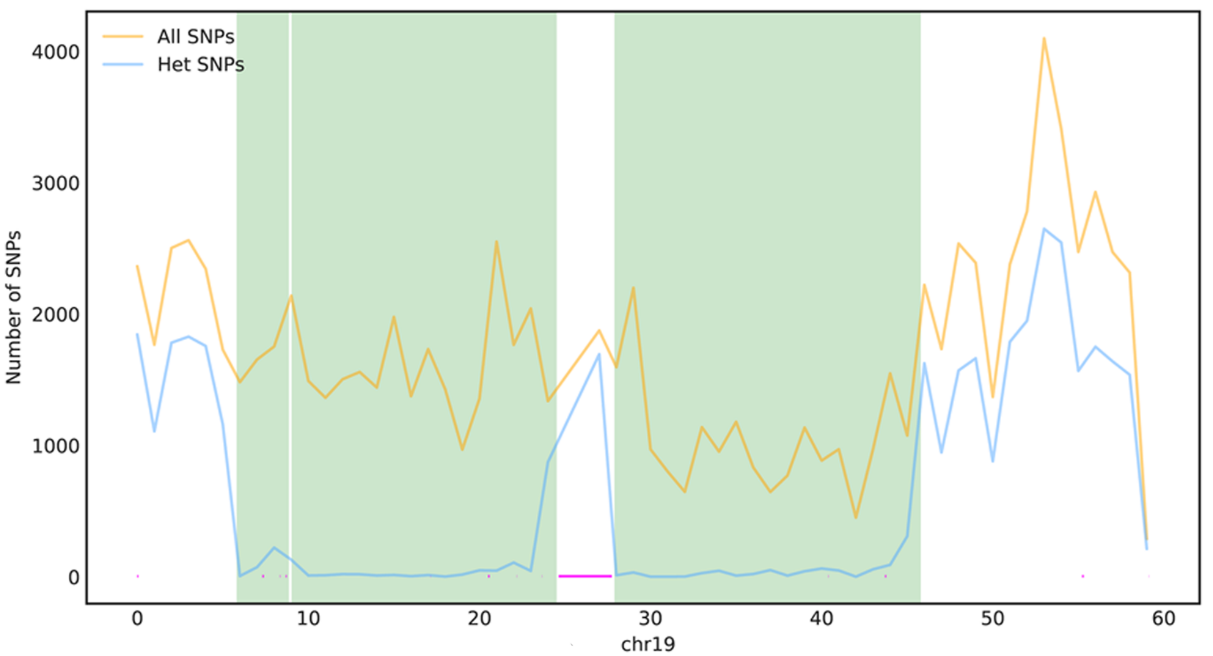
 **Supplementary Figure 27.** WGS results for chromosome 19 in NA00338. Three AOH regions highlighted in light green were identified on chromosome 19 in NA00338 as following: seq[GRCh37] hmz(19)(p13.3q13.32) chr19:g.5813244_8863379hmz; hmz(19)(p13.3q13.32) chr19:g.9022351_24507463hmz; hmz(19)(p13.3q13.32) chr19:g.27889966_45787566hmz.


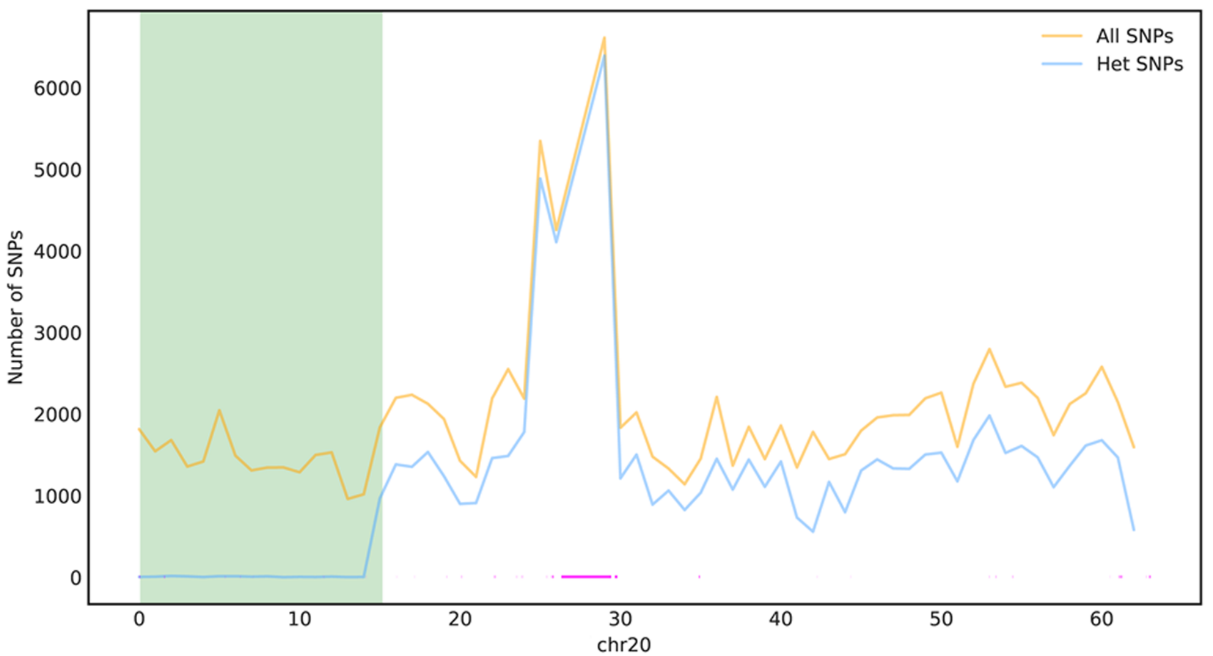


**Supplementary Figure 28.** WGS results for chromosome 20 in NA00338. One AOH region highlighted in light green was identified on chromosome 20 in NA00338 as following: seq[GRCh37] hmz(20)(p13p12.1)chr20:g.65900_15168170hmz.


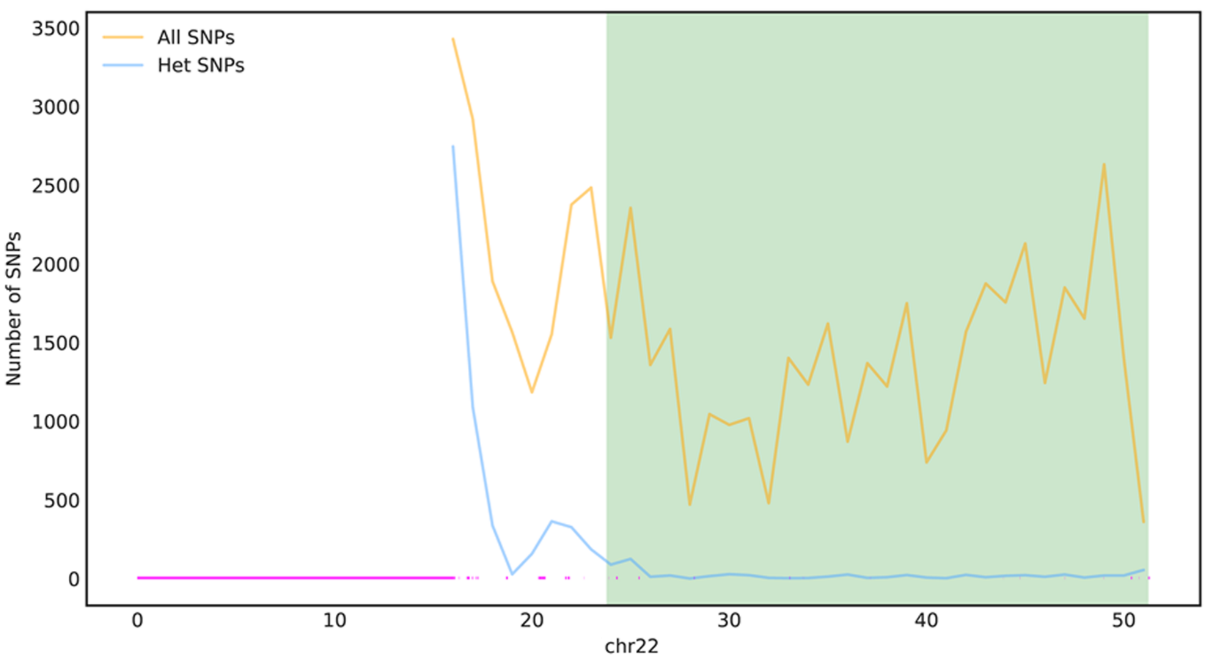


**Supplementary Figure 29.** WGS results for chromosome 22 in NA00338. One AOH region highlighted in light green was identified on chromosome 22 in NA00338 as following: seq[GRCh37] hmz(22)(q11.1q13.33) chr22:g.23778112_51242823hmz.

## Supplementary Tables

**Supplementary Table 1.** WGS results for 45 cases with known variants.

| **Sample name** | **Source of the sample** | **Library construction** | **Platform** | **Known variant type** | **Known variants** | **Verification methods** | **WGS results** |
| --- | --- | --- | --- | --- | --- | --- | --- |
| Case 1 | Laboratory-held positive cell line | PCR free | MGISEQ2000, PE150 | SNV | *DMD* c.5009G>A p.Trp1670*, hemi | Sanger | NM_004006.2(*DMD*):c.5009G>A(p.Trp1670*), hemi |
| Case 2 | Laboratory-held positive cell line | PCR free | MGISEQ2000, PE150 | SNV | *HBB* IVS-II-654,-α^4.2^/-α^4.2^ | Sanger | NM_000518.4(*HBB*): c.316-197C>T, het |
| Case 3 | Laboratory-held positive cell line | PCR free | MGISEQ2000, PE150 | SNV | *DMD* c.1997C>A p.Ser666*,hemi | Sanger | NM_004006.2(*DMD*):c.1997C>A(p.Ser666*), hemi |
| NA09912 | Coriell | PCR free | MGISEQ2000, PE150 | SNV | *CYP2D6*, IVSDS3, G>A, +1 | Sanger | NM_000106.5(*CYP2D6*): c.506-1G>A, hom |
| Case 4 | Laboratory-held positive cell line | PCR free | MGISEQ2000, PE150 | Indel | *DMD* c.2959delA p.Ser987fs, hemi | Sanger | NM_004006.2(*DMD*):c.2959delA(p.Ser987Valfs*17), hemi |
| Case 5 | Laboratory-held positive cell line | PCR free | MGISEQ2000, PE150 | Indel | *DMD* c.8098_8099delAA p.Lys2700Valfs*9, hemi | Sanger | NM_004006.2(*DMD*):c.8098_8099delAA(p.Lys2700Valfs*9), hemi |
| Case 6 | Clinical case from Pu'er People's Hospital | PCR free | MGISEQ2000, PE150 | MT variants | *MT-TL1*: m.3243A>G | Sanger | NC_012920.1(*MT-TL1*): m.3243A>G, 19% heteroplasmy |
| Case 7 | Clinical case from Pu'er People's Hospital | PCR free | MGISEQ2000, PE150 | MT variants | *MT-RNR1*: m.1555A>G | Sanger | NC_012920.1(*MT-RNR1*): m.1555A>G, 10% heteroplasmy |
| Case 8 | Clinical case from Pu'er People's Hospital | PCR free | MGISEQ2000, PE150 | MT variants | *MT-TL1*: m.3243A>G | Sanger | NC_012920.1(*MT-TL1*): m.3243A>G, 41% heteroplasmy |
| Case 9 | Clinical case from Pu'er People's Hospital | PCR free | MGISEQ2000, PE150 | Aneuploid | 47,XY,+18 | Karyotyping | seq[GRCh37] (18)x3 |
| Case 10 | Clinical case from Pu'er People's Hospital | PCR free | MGISEQ2000, PE100 | Aneuploid | 47,XX,+13 | Karyotyping | seq[GRCh37] (13)x3 |
| Case 11 | Clinical case from Pu'er People's Hospital | PCR free | MGISEQ2000, PE100 | Aneuploid | 45,X | Karyotyping | seq[GRCh37] (X)x1 |
| Case 12 | Clinical case from Pu'er People's Hospital | PCR free | MGISEQ2000, PE100 | Aneuploid | 47,XYY | Karyotyping | seq[GRCh37] (X)x1,(Y)x2 |
| NA12722 | Coriell | PCR free | MGISEQ2000, PE100 | Aneuploid (Chimerism) | 46,XY,del(9)(p13.3p13.1)[25] 48,XY,+9,del(9)(p13.3p13.1),+18 [16] 47,XY,del(9)(p13.3p13.1),+18[9] arr 9p13.3p13.1(33406379-39305024)x1 | Karyotyping, CMA | seq[GRCh37] (9,18)x3 del(9)(p13.3p13.1) chr9:g.33416001_40991000del |
| Case 13 | Clinical case from Pu'er People's Hospital | PCR free | MGISEQ2000, PE100 | Triploid | 69,XXX | Karyotyping | seq[GRCh37] (X,1-22)x3 |
| Case 14 | Laboratory-held positive cell line | PCR free | MGISEQ2000, PE100 | CNV | *DMD* EXON 3-44 Del, het | qPCR | seq[GRCh37] del(X)(p21.1) chrX:g.32212001_32960000del |
| ID00016 | Coriell | PCR free | MGISEQ2000, PE100 | CNV | DIGEORGE SYNDROME | NA | seq[GRCh37] del(22)(q11.21) chr22:g.18885001_21490000del |
| Case 15 | Laboratory-held positive cell line | PCR free | MGISEQ2000, PE100 | CNV | del(17p13.3, 2.10M) | Low pass WGS | seq[GRCh37] del(17)(p13.3) chr17:g.1_2089000del |
| Case 16 | Laboratory-held positive cell line | PCR free | MGISEQ2000, PE100 | CNV | del(1p36.33p36.32,2.33M) | Low pass WGS | seq[GRCh37] del(1)(p36.33p36.32) chr1:g.1_2583000del |
| Case 17 | Laboratory-held positive cell line | PCR free | MGISEQ2000, PE100 | CNV | *DMD* EXON 46-50 Del, het | qPCR | seq[GRCh37] del(X)(p21.1) chrX:g.31806001_31984000del |
| Case 18 | Laboratory-held positive cell line | PCR free | MGISEQ2000, PE100 | CNV | *DMD* EXON 3-44 Del. Hom | qPCR | seq[GRCh37] del(X)(p21.1) chrX:g.32212001_32959000del |
| Case 19 | Laboratory-held positive cell line | PCR free | MGISEQ2000, PE100 | CNV | del(15q11.2q13.1,5.2M) | Low pass WGS | seq[GRCh37] del(15)(q11.2q13.1) chr15:g.23605001_28547000del |
| Case 20 | Laboratory-held positive cell line | PCR free | MGISEQ2000, PE100 | CNV | del(7q11.23,1.48M) | Low pass WGS | seq[GRCh37] del(7)(q11.23) chr7:g.72682001_74143000del |
| Case 21 | Laboratory-held positive cell line | PCR free | MGISEQ2000, PE100 | CNV | *DMD* EXON 18-20 Del, het | qPCR | seq[GRCh37] del(X)(p21.1) chrX:g.32509001_32538000del |
| Case 22 | Laboratory-held positive cell line | PCR free | MGISEQ2000, PE100 | CNV | *DMD* EXON 44 Del, het | qPCR | seq[GRCh37] del(X)(p21.1) chrX:g.32100001_32264000del |
| Case 23 | Laboratory-held positive cell line | PCR free | MGISEQ2000, PE100 | CNV | *DMD* EXON 45 Del, het | qPCR | seq[GRCh37] del(X)(p21.1) chrX:g.31950001_32055000del |
| NA17235 | Coriell | PCR free | MGISEQ2000, PE100 | CNV | Heterozygous deletion of the *CYP2D6* locus | long range PCR | seq[GRCh37] del(22)(q13.2) chr22:g.42521001_42532000del |
| NA19317 | Coriell | PCR free | MGISEQ2000, PE100 | CNV | Homozygous deletion of the *CYP2D6* locus | long range PCR | seq[GRCh37] del(22)(q13.2)x2  NC_000022.10:g.[42519001_42534000del];[42519001_42534000del] |
| Case 24 | Laboratory-held positive cell line | PCR free | MGISEQ2000, PE100 | CNV | *DMD* EXON 45-48 Del, het | qPCR | seq[GRCh37] del(X)(p21.1) chrX:g.31870001_32018000del |
| Case 25 | Laboratory-held positive cell line | PCR free | MGISEQ2000, PE100 | CNV | *DMD* EXON 49-52 Del, het | qPCR | seq[GRCh37] del(X)(p21.1) chrX:g.31747001_31857000del |
| NA09912 | Coriell | PCR free | MGISEQ2000, PE100 | CNV | Heterozygous deletion of the *CYP2D6* locus | long range PCR | seq[GRCh37] del(22)(q13.2) chr22:g.42521001_42532000del |
| NA17244 | Coriell | PCR free | MGISEQ2000, PE100 | CNV | Duplication of the *CYP2D6* locus | long range PCR | seq[GRCh37] dup(22)(q13.2) chr22:g.42520001_42534000dup |
| GM24302 | Coriell | PCR free | MGISEQ2000, PE100 | CNV | 46,XY,del(17)(p11.2p11.2).arr[hg19] 17p11.2(16735531-20373037)x1 | CMA | seq[GRCh37] del(17)(p11.2) chr17:g.16749001_20416000del |
| Case 26 | Laboratory-held positive cell line | PCR free | MGISEQ2000, PE100 | CNV | *DMD* EXON 48-50 Dup, het | qPCR | seq[GRCh37] dup(X)(p21.1) chrX:g.31832549_31903740dup |
| GM01063 | Coriell | PCR free | MGISEQ2000, PE100 | Balanced translocation | 46,XX,t(4;13)(q31.3;q14.3) | Karyotyping | seq[GRCh37] t(4;13)(q32.2(161913247);q21.1(59345836)) |
| Case 27 | Laboratory-held positive cell line | PCR free | MGISEQ2000, PE100 | Balanced translocation | 46,XY,t(8;18)(p21;p11.2) | Karyotyping | seq[GRCh37] t(8;18)(p22(17735160_17735170);p11.31(4262857_4262862)) |
| Case 28 | Laboratory-held positive cell line | PCR free | MGISEQ2000, PE100 | Balanced translocation | 46,XX,t(5;7)(p15.1;q32) | Karyotyping | seq[GRCh37] t(5;7)(p14.1(26035448);q31.31(120254553)) |
| Case 29 | Laboratory-held positive cell line | PCR free | MGISEQ2000, PE100 | Balanced translocation | 46,XX,t(6;11)(p23;p13) | Karyotyping | seq[GRCh37] t(6;11)(p25.1(4984417_4986257);p14.3(25872804_25872824)) |
| GM24264 | Coriell | PCR free | MGISEQ2000, PE150 | Balanced translocation | 46,XX,t(1;3)(q32.1;q25.2) | Karyotyping | seq[GRCh37] t(1;3)(q32.1(210192903_210192913);q25.2(153609776_153609774)) |
| GM10797 | Coriell | PCR free | MGISEQ2000, PE100 | Thalassemia CNV | --^SEA^/--^FIL^ | gap-PCR | --^SEA^/--^FIL^ |
| Case 2 | Laboratory-held positive cell line | PCR free | MGISEQ2000, PE100 | Thalassemia CNV | *HBB* IVS-II-654,-α^4.2^/-α^4.2^ | gap-PCR | -α^4.2^/-α^4.2^ |
| GM21081 | Coriell | PCR free | MGISEQ2000, PE100 | Thalassemia CNV | Filipino beta-O-thal deletion | gap-PCR | seq[GRCh37] del(11)(p15.34) chr11:g.5134001_5253000del |
| Case 30 | Laboratory-held positive cell line | PCR free | MGISEQ2000, PE100 | Thalassemia CNV | αα/-α^4.2^ | gap-PCR | αα/-α^4.2^ |
| NA00338 | Coriell | PCR free | MGISEQ2000, PE100 | AOH | 46,XY[24].arr[hg19] (1p31.1p22.2(79,672,537-88,410,647),1q23.2q41(160,492,269-222,339,638),3p14.1q25.1(68,583,520-151,160,648),4q13.3q35.2(72,417,647-188,486,074),5p15.32p13.3(4,932,612-31,756,213),5q14.3q33.2(82,871,756-155,086,996),5q35.2q35.3(174,136,120-180,692,833),6p25.3p25.1(203,248-5,893,280),6p22.3q15(16,424,116-91,531,365),7p22.3p14.3(43,258-33,772,969),7q21.12q36.3(87,462,833-155,241,247),9p24.3p23(46,586-9,863,227),10p13p11.22(13,030,205-31,909,119),10q25.1q26.2(110,761,069-128,459,850),13q14.12q33.2(45,630,007-104,867,785),13q33.3q34(107,656,460-115,106,996),14q12q23.2(25,913,873-64,343,703),15q11.2q15.1(23,855,238-40,617,414),16p13.3q12.1(91,009-49,565,261),17p13.3p13.1(6,688-7,580,052),17q12q25.3(37,092,287-78,513,216),18p11.31p11.21(5,829,906-15,215,871),19p13.3q13.32(5,839,977-45,785,193),20p13p12.1(61,794-15,447,711), 22q11.1q13.33(16,055,170-51,219,006))x2 hmz | CMA | seq[GRCh37] hmz(1)(p31.1p22.2)chr1:g.79638609_85981487hmz,hmz(1)(q23.2q41)chr1:g.160474013_206309249hmz,hmz(1)(q23.2q41)chr1:g.206566766_222379937hmz,hmz(3)(p24.2p24.1)chr3:g.25643989_28680907hmz,hmz(3)(p14.1q25.1)chr3:g.68571007_75270810hmz,hmz(3)(p14.1q25.1)chr3:g.77835431_90450435hmz,hmz(3)(p14.1q25.1)chr3:g.93509006_129799485hmz,hmz(3)(p14.1q25.1)chr3:g.129809474_151171901hmz,hmz(4)(q13.3q35.2)chr4:g.72346616_132653117hmz,hmz(4)(q13.3q35.2)chr4:g.132671346_188485278hmz,hmz(5)(p15.32p13.3)chr5:g.4913484_21082817hmz,hmz(5)(p15.32p13.3)chr5:g.21572713_29425215hmz,hmz(5)(p15.32p13.3)chr5:g.29434245_31702283hmz,hmz(5)(q14.3q33.2)chr5:g.82852908_155197081hmz,hmz(5)(q35.2q35.3)chr5:g.177348614_180903190hmz,hmz(6)(p25.3p25.1)chr6:g.360038_5910305hmz,hmz(6)(p22.3q15)chr6:g.16475066_26727284hmz,hmz(6)(p22.3q15)chr6:g.26775039_32440405hmz,hmz(6)(p22.3q15)chr6:g.32728902_57206220hmz,hmz(6)(p22.3q15)chr6:g.61920506_91544177hmz,hmz(7)(p22.3p14.3)chr7:g.550944_33777808hmz,hmz(7)(q21.12q36.3)chr7:g.87533090_100546815hmz,hmz(7)(q21.12q36.3)chr7:g.102314531_142047342hmz,hmz(7)(q21.12q36.3)chr7:g.144048199_149725582hmz,hmz(7)(q21.12q36.3)chr7:g.149744605_151935567hmz,hmz(9)(p24.3p23)chr9:g.31589_9866177hmz,hmz(10)(p13p11.22)chr10:g.13027881_26860043hmz,hmz(10)(q25.1q26.2)chr10:g.27621971_31916276hmz,hmz(10)(q25.1q26.2)chr10:g.110757671_127577066hmz,hmz(13)(q14.12q33.2)chr13:g.45607568_63603241hmz,hmz(13)(q33.3q34)chr13:g.63648556_104875972hmz,hmz(13)(q33.3q34)chr13:g.107654799_115109706hmz,hmz(14)(q12q23.2)chr14:g.25888146_64349064hmz,hmz(15)(q11.2q15.1)chr15:g.23851100_28538769hmz,hmz(15)(q11.2q15.1)chr15:g.28957120_40641447hmz,hmz(16)(p13.3q12.1)chr16:g.60291_14972915hmz,hmz(16)(p13.3q12.1)chr16:g.22671215_28531357hmz,hmz(16)(p13.3q12.1)chr16:g.28737086_32006758hmz,hmz(17)(p13.3p13.1)chr17:g.396660_7584400hmz,hmz(17)(q12q25.3)chr17:g.37145222_41408858hmz,hmz(17)(q12q25.3)chr17:g.43662883_78528340hmz,hmz(18)(p11.31p11.21)chr18:g.5812234_14182114hmz,hmz(19)(p13.3q13.32)chr19:g.5813244_8863379hmz,hmz(19)(p13.3q13.32)chr19:g.9022351_24507463hmz,hmz(19)(p13.3q13.32)chr19:g.27889966_45787566hmz,hmz(20)(p13p12.1)chr20:g.65900_15168170hmz,hmz(22)(q11.1q13.33)chr22:g.23778112_51242823hmz |
| NA04648 | Coriell | PCR free | MGISEQ2000, PE100 | Repeat expansions | *DMPK* CTG expansion repeat alleles are normal and approximately 1000 | PCR | *DMPK* CTG repeat expansion 5/62^a^ |
| NA13717 | Coriell | PCR free | MGISEQ2000, PE100 | Repeat expansions | *DRPLA(ATN1)* CAG expansion repeat alleles are 15 and 65 | PCR | *ATN1* CAG repeat expansion 15/55 |
| Case 31 | Laboratory-held positive cell line | PCR free | MGISEQ2000, PE100 | SMA | *SMN1* EXON7-8 Del, hom | MLPA | seq[GRCh37] del(5)(q13.2)x2 chr5:g.(?_70247768)_(70248842_?)del |

^a^For Case NA04648, the difference between the results of WGS and PCR is due to the limitation of ExpansionHunter in its sizing of alleles considerably larger than the read length [4].

**Supplementary Table 2.** The number of variants and time required for manual interpretation of each type of variant for one sample.

| Types of variants | The average number of variants required for manual interpretation after bioinformatics analysis | The average time required for manual interpretation |
| --- | --- | --- |
| SNV/INDEL | 300-400 | 4 hours |
| Noncoding variant | 10-30 |  |
| MT variants | 5-20 | 30 minutes |
| CNV | 30-50 | 1 hour |
| SV (balanced translocation and inversion) | 20-30 | 30 minutes |
| Thalassemia CNV | 0-1 (auto) | 0 |
| AOH | 0-1 | 30 minutes |
| SMA (EXON7-8 Del) | 0-1(auto) | 0 |
| Repeat expansion | 0-1 (auto) | 0 |
| Intrauterine infection | 0-1 (auto) | 0 |

**References**

1. Jian X, Boerwinkle E, Liu X. In silico prediction of splice-altering single nucleotide variants in the human genome. Nucleic Acids Res. 2014;42(22):13534-44.

2. Leman R, Gaildrat P, Le Gac G, Ka C, Fichou Y, Audrezet MP, et al. Novel diagnostic tool for prediction of variant spliceogenicity derived from a set of 395 combined in silico/in vitro studies: an international collaborative effort. Nucleic Acids Res. 2018;46(15):7913-23.

3. Robinson JT, Thorvaldsdottir H, Wenger AM, Zehir A, Mesirov JP. Variant Review with the Integrative Genomics Viewer. Cancer Res. 2017;77(21):e31-e4.

4. Ibanez K, Polke J, Hagelstrom RT, Dolzhenko E, Pasko D, Thomas ERA, et al. Whole genome sequencing for the diagnosis of neurological repeat expansion disorders in the UK: a retrospective diagnostic accuracy and prospective clinical validation study. Lancet Neurol. 2022;21(3):234-45.
